# Absence of sympathetic innervation hampers the generation of tertiary lymphoid structures upon acute lung inflammation

**DOI:** 10.1038/s41598-024-62673-0

**Published:** 2024-05-23

**Authors:** Clémence Riffard, Laïla Letaïef, Safa Azar, Armanda Casrouge, Isabelle Brunet, Jean-Luc Teillaud, Marie-Caroline Dieu-Nosjean

**Affiliations:** 1 Faculté de Santé Sorbonne Université, Sorbonne Université UMRS1135, 75013 Paris, France; 2 Inserm U1135, 75013 Paris, France; 3 Laboratory “Immune Microenvironment and Immunotherapy”, Centre of Immunology and Microbial Infections, CIMI Paris, 75013 Paris, France; 4 Center for Interdisciplinary Research in Biology (CIRB), Collège de France, CNRS UMR7241, Inserm U1050, 75231 Cedex Paris, France; 5 Université Paris Sciences and Lettres, 75231 Cedex Paris, France

**Keywords:** 6-OHDA, Acute lung inflammation, B cell, Lymphoid aggregate, Sympathetic nervous system, Tertiary lymphoid structure, Neuroscience, Imaging, Immunology, Inflammation

## Abstract

Tertiary lymphoid structures (TLS) are lymphoid organs present in inflammatory non-lymphoid tissues. Studies have linked TLS to favorable outcomes for patients with cancers or infectious diseases, but the mechanisms underlying their formation are not fully understood. In particular, secondary lymphoid organs innervation raises the question of sympathetic nerve fibers involvement in TLS organogenesis. We established a model of pulmonary inflammation based on 5 daily intranasal instillations of lipopolysaccharide (LPS) in immunocompetent mice. In this setting, lung lymphoid aggregates formed transiently, evolving toward mature TLS and disappearing when inflammation resolved. Sympathetic nerve fibers were then depleted using 6-hydroxydopamine. TLS quantification by immunohistochemistry showed a decrease in LPS-induced TLS number and surface in denervated mouse lungs. Although a reduction in alveolar space was observed, it did not impair overall pulmonary content of transcripts encoding TNF-α, IL-1β and IFN-γ inflammation molecules whose expression was induced by LPS instillations. Immunofluorescence analysis of immune infiltrates in lungs of LPS-treated mice showed a drop in the proportion of CD23^+^ naive cells among CD19^+^ B220^+^ B cells in denervated mice whereas the proportion of other cell subsets remained unchanged. These data support the existence of neuroimmune crosstalk impacting lung TLS neogenesis and local naive B cell pool.

## Introduction

Tertiary lymphoid structures (TLS) are ectopic lymphoid aggregates that transiently develop in non-lymphoid tissue upon proinflammatory stimuli. They are observed in chronic infections, autoimmune diseases, graft rejection or cancers, where they are associated with an exacerbation of immune responses, with a good or bad prognostic value depending on the disease^1–4^. Although the role of TLS has been extensively investigated, the mechanisms underlying their formation and function are still only partially elucidated.

Sympathetic fibers are present in lymph nodes^5^ and are involved in the regulation of the immune response through the production of catecholamines that bind β2-adrenergic receptors and D1-like receptors expressed by immune cells^6,7^. These neurotransmitters are involved in inhibitory or activating mechanisms. For instance, in vitro stimulation by catecholamines of β-adrenergic receptors and BCR/TLR9 expressed by B cells inhibits CD4^+^ T cells^8^ and impairs immune responses against infectious pathogens^9,10^. Conversely, they can oppose immune suppression. In a murine model of experimental autoimmune encephalomyelitis (EAE)**,** the depletion of sympathetic nerve fibers by the catecholaminergic neurotoxin 6-hydroxydopamine (6-OHDA) provoked an increase in CD4^+^ FoxP3^+^ regulatory T cells (Tregs) in lymph nodes and spleen^11^. Similarly, mice unable to produce noradrenaline had impaired T cell responses when infected with *L. monocytogenes* or *M. tuberculosis*, indicating that noradrenaline impacts T-cell-driven immunity^12^.

The involvement of sympathetic nerve fibers in the function of secondary lymphoid organs and their role in the formation of arterial plaque-associated lymphoid structures have been investigated in detail. 6-OHDA-induced sympathetic denervation and the subsequent drop in noradrenaline caused the collapse of these lymphoid structures^13^. Nerve fibers have also been shown to colocalize with lymphoid aggregates, mostly B cells, in the pancreatic cancer microenvironment. In that case, high nerve fiber density correlated with better survival of patients presenting a high density of lymphoid aggregates^14^. Thus, understanding to what extent the sympathetic nervous system influences TLS formation has now become of growing interest. It should be stressed that the absence of a fibrous capsule around TLS represents another challenge for the aggregation and maintenance of the structures in which sympathetic nerve fibers might be involved.

In the present study, we used a murine model relying on repeated intranasal (i.n.) administration of lipopolysaccharide (LPS) to investigate the involvement of the sympathetic nervous system in TLS formation following acute pulmonary inflammation. We show that LPS instillations induce a transient formation of lymphoid aggregates that become mature TLS and disappear upon resolution of acute inflammation. 6-OHDA-induced depletion of sympathetic fibers resulted in a significant decrease in TLS density and number following LPS treatment, although it did not fully abolish their formation. Sympathetic denervation also led to a drop in the proportion of naive B cells in the lungs of LPS-treated mice. Thus, we conclude that crosstalk between sympathetic nerve fibers and immune cells impacts TLS neogenesis and the local naive B cell pool in inflamed lung tissue.

## Methods

### Mice

Eight- and 12-week-old female C57BL/6 mice were purchased from Janvier laboratories (Le Genest-Saint-Isle, France) and kept under pathogen-free conditions at the animal facility (UMS28, School of Medicine, Sorbonne University, Paris, France). Mice were housed in the facility at least seven days prior to any experimentation, and all animal studies were performed in compliance with the European guidelines and with the approval of the Charles Darwin Ethics Committee for animal experimentation (Paris, France) (agreement APAFIS#23442-2019122316202171 v4). Animals were euthanized by cervical dislocation, and blood and tissues (brain, kidney, spleen, lungs, ears) were retrieved.

### LPS intranasal instillations

Thirteen-week-old female C57BL/6 mice were anesthetized with a ketamine/xylazine solution (ketamine 8%, 100 mg/kg, Virbac, Carros, France; xylazine 0.4%, 16 mg/kg, Bayer, La Garenne-Colombes, France, in NaCl 0.9%) administered intraperitoneally (i.p.) (0.1 mL). Then, either LPS (Sigma‒Aldrich, Saint-Louis, MO, Cat# L4130) or NaCl 0.9% solutions were dripped onto the surface of the mouse's nostril using a 20 µL pipette and filtered cones (14 µL into each nostril, 10 µg total LPS/animal) each day for 5 consecutive days.

### Sympathetic nerve fiber depletion by 6-hydroxydopamine (6-OHDA)

Nine-week-old female C57BL/6 mice were administered i.p. NaCl 0.9% or 6-OHDA solutions (Sigma‒Aldrich, Cat# 162957), as previously described in the literature^15^. 1.90 mg 6-OHDA in 200 µL NaCl/mouse were injected, and 4.75 mg 6-OHDA in 200 µL NaCl/mouse two days later.

### Whole mount staining of ears

Ears were separated, and only the part showing vasculature was kept and fixed in 4% PFA for 30 min. After three washes in PBS, nonspecific binding was blocked by incubation of the ears in 0.1 M Tris–HCl, 0.3 M NaCl, blocking buffer (Perkin Elmer) (pH 7.4) containing 0.5% Triton X-100 (TBNT) for 4 h at RT. The anti-TH antibody (Merck Millipore, Saint-Quentin-en-Yvelines, France, Cat# AB152) was then added and incubated overnight at 4 °C. Anti-α-SMA-Cy3 antibody (Sigma‒Aldrich, Cat# C6198) and donkey anti-rabbit IgG-AF647 (Invitrogen, Carlsbad, CA, USA, Cat# A-31573) were then incubated for 4 h after at least three washes with 0.1 M Tris–HCl, 0.3 M NaCl (pH 7.4) containing 0.5% Triton X-100 (TNT). Ears were mounted in Dako fluorescent medium (Agilent Technologies, Cat# S3023), and images were acquired with the Zeiss Axioimager Z2 Apotome (Carl Zeiss Microscopy, Jena, Germany). The antibodies used are listed in Supplementary Table S1.

### Quantification of catecholamine plasmatic concentration

Mouse blood was collected in heparin tubes and then centrifuged (5 min, 2000×*g*, RT). Plasma was then kept at − 20 °C before use. Catecholamine concentration in 100 µL plasma samples was determined using an ELISA kit (Antibodies-Online, Aachen, Germany, Cat# ABIN772979), as recommended by the manufacturer. O.D._450 nm_ reading was then performed.

### Western blot

Proteins were extracted from the brain, kidney and lungs of 6-OHDA- and NaCl-treated mice. Protein concentration was assessed with a BCA Protein Assay Kit (Merck Millipore, Cat# 71285-3). Thirty micrograms of protein/lane were separated by SDS‒PAGE (4–15% Mini-Protean TGX Precast protein gel) (Bio-Rad, Hercules, CA, USA, Cat# 4561086). Gels were then blotted onto 0.2 μm PVDF membranes (Bio-Rad, Cat# 1620177) and blocked with 5% dried milk in TBST (Tris-buffered saline, 0.1% Tween 20) for 2 h. Membranes were incubated overnight with anti-TH or anti-GAPDH antibody at 4 °C. After washing with TBST, the membranes were incubated with peroxidase-conjugated revealing antibodies for 1 h at RT. Protein detection was carried out by chemiluminescence (Clarity Western ECL substrate kit) (Bio-Rad, Cat# 1705060). Data were captured with the Fusion FX imaging system (Vilber, Marne-la-Vallée, France). The antibodies used in this section are listed in Supplementary Table S1.

### Reverse transcription and real-time quantitative PCR

Total RNA was extracted from mouse lungs using Purelink RNA Mini Kit (Thermo Fisher, Cat #12183018A) according to manufacturer’s recommendations, and reverse transcription was performed with TaqMan™ Reverse Transcription Reagents (Thermo Fisher, Cat #N8080234). Real-time quantitative PCR was then carried out using TaqMan™ gene expression assay (Thermo Fisher, Cat #4453320) and TaqMan™ Fast Advanced Master Mix (Thermo Fisher, Cat #4444963) and a QuantStudio 3 analyzer (Applied Biosystems). *Actb* gene was used as endogenous control for each sample. *Actb*, *Tnfa*, *Il1b* and *Ifng* gene expression were tested in duplicates. Data were analysed using Data Design and Analysis Desktop Software (Applied Biosystems, v2.8.0).

### Spleen and lung cell analysis by flow cytometry

Spleens and lungs from 6-OHDA- and NaCl-treated mice were harvested at day 30 and day 40 posttreatment. They were digested with collagenase A (Interchim, Cat# 10103586001) (250 units/mL in RPMI-1640), dissociated using a GentleMACS Octo Dissociator (Miltenyi, Cat# 130-096-427), passed through a 70 µm pore size filter (lungs) or manually shredded directly on the filter (spleen). Red blood cells were then lysed in ACK lysis buffer (154.4 mM NH_4_Cl, 10 mM KHCO_3_) for 1 min at room temperature. The reaction was then stopped by adding Wash Buffer (PBS, 5% FCS, 0.5 mM EDTA). Cells were centrifuged (10 min, 200×*g*, 4 °C), resuspended in PBS containing 2% FCS at 2 × 10^7^/mL and distributed into a 96-well conical bottom plate (100 μL/well). FcγRII (CD32) and FcγRIII (CD16) were saturated by incubating cells for 20 min at 4 °C with 2.4G2 antibody (produced in the laboratory). After centrifugation, antibody mixes (Supplementary Table S2) were added and incubated in the dark at 4 °C for 20 min. After centrifugation and washing, cells were fixed in PBS containing and 1% PFA in the dark at 4 °C for 10 min. After a final wash, cells were transferred into tubes for flow cytometry analysis (Fortessa 5 lasers, Beckton Dickinson, Pont-de-Claix, France).

### Hematoxylin and eosin tissue counterstaining

Sixty 4 µm-thick frontal sections were obtained from each formalin-fixed paraffin-embedded (FFPE) lung, as to cover the whole organ volume (a few outer sections showing incomplete lobes were discarded), and deposited on Superfrost slides. One every four sections was used for hematoxylin and eosin (H&E) counterstaining and further TLS quantification. Slides were first dewaxed in two Clearene baths (3 min each) (Leica Biosystems, Buffalo Grove, IL, USA; Cat# 3803600) and rehydrated in successive 3 min baths in graded ethanol (100%, 90%, 70%, 50%, and 0%). They were then immersed in hematoxylin (Dako, Agilent Technologies, les Ulis, France; Cat# CS700) for 1 min, rinsed in tap water and immersed again in eosin for 45 s (Thermo Fisher, Cat# 71204). After several rinses in distilled water, slides were mounted using Glycergel® mounting medium (Dako, Agilent Technologies, Cat# C056330-2) previously heated to 50 °C. Slides were then kept at 4 °C. Scanning of single plane images was performed with a Nanozoomer (Hamamatsu, Massy, France). White balance was adjusted on the final images so that areas without tissue remained white.

### Quantification of lung lymphoid aggregates and TLS

Images obtained from the scanning of H&E slides were analyzed with HALO v3.3.2541.345 (IndicaLabs, Albuquerque, NM, USA). The DenseNet (Dense Convolutional Network) artificial intelligence module was trained to recognize all types of lymphoid aggregates, including TLS (LA-TLS) and pulmonary tissue areas on lung sections. Each slide was assessed manually, and quantification of surfaces and number of aggregates was performed by HALO software. LA-TLS density was calculated as the ratio of the LA-TLS surface to the lung tissue surface multiplied by 100. The lung tissue surface was determined either including the alveolar surface or not. LA-TLS numbers were expressed by mm^2^ of tissue section.

### Immunofluorescence staining

FFPE lung sections were dewaxed and rehydrated as described above with 5 min baths. They were then immersed in citrate buffer pH 6.0 (Dako, Agilent Technologies, Cat# S1699) or pH 9.0 (Dako, Agilent Technologies, Cat# S2397) for antigen retrieval for 30 min at 97 °C. Endogenous peroxidase was inhibited by incubation in 3% H_2_O_2_ for 30 min. After washing, lung sections were incubated in Dako Protein block reagent (Dako, Agilent Technologies, Cat# X090930-2) for 30 min. Primary antibody staining was performed for 1 h at RT. Tissue sections were then incubated with secondary HRP-conjugated antibody for 30 min at RT, and fluorescently labeled tyramide polymers were used as a substrate for the HRP reaction. To remove bound antibodies, slides were immersed again in citrate buffer pH 6.0 at 97 °C for 10 min, cooled and washed in TBS. Slides were then blocked again for 30 min using Dako Protein block reagent before starting a new staining cycle (primary antibody + secondary antibody-HRP + fluorescence-coupled tyramide). This multiplex technique was performed with the antibodies and reagents listed in Supplementary Table S1. Cell nuclei were stained with 1 µg/mL DAPI (Thermo Fisher, Cat# 62248). Slides were then mounted using Fluorescence Mounting Medium (Dako, Agilent Technologies, Cat# S3023) and kept at 4 °C. Acquisition of single plane images was performed with a confocal fluorescence microscope (Carl Zeiss Microscopy, Zen 3.3.89.0000 software).

### iDISCO (immunolabeling-enabled three-dimensional imaging of solvent-cleared organs)

Lungs were fixed in 4% PFA, dehydrated in graded methanol (20%, 40%, 60%, 80%, and 100%, each for 1 h at RT), washed twice in 100% methanol and cooled at 4 °C. The lungs were then incubated in 66% dichloromethane (DCM) 33% methanol overnight under agitation at 4 °C and washed again twice in 100% methanol at 4 °C (1 h each). Lungs were then bleached in 5% H_2_O_2_ overnight at 4 °C, rehydrated with graded methanol (80%, 60%, 40%, and 20%, each for 1 h at RT) and washed twice in PTx.2 (0.01 M PBS, 0.2% Triton X-100, 1 h each) at RT. Permeabilization was then carried out for two days at 37 °C under agitation with 0.01 M PBS, 0.2% Triton X-100, 20% DMSO, 0.3 M glycine. Nonspecific binding sites were blocked with 0.2% Triton X-100, 10% DMSO, and 5% donkey serum by incubation for two days at 37 °C under agitation. Following blocking, lungs were incubated with anti-TH antibody in 0.01 mM PBS, 0.2% Tween-20, 10 mg/mL heparin, 5% DMSO, and 3% heat-inactivated donkey serum for 3 days at 37 °C under agitation. The lungs were then washed several times and incubated overnight in 0.01 mM PBS, 0.2% Tween-20, and 10 mg/mL heparin (PTwH) before adding anti-α-SMA-Cy3 antibody (Sigma‒Aldrich) and donkey anti-rabbit IgG-AF647 antibody (Invitrogen) for 3 days at 37 °C under agitation. After staining, the lungs were washed several times with PTwH and incubated overnight in PTwH at RT. For clearing, lungs were dehydrated in graded methanol, incubated again in 66% DCM for 3 h at RT and washed twice in 100% DCM before storage in di-benzyl-ether. All the antibodies used in this section are listed in Supplementary Table S1.

### Imaging for 3D staining

All samples were analyzed with a light sheet microscope (Ultramicroscope, LaVision BioTec, Bielefeld, Germany). Excitation laser wavelengths of 561 and 647 nm were used. A Z-step size of 3 µm was used for lung imaging. All data were collected in a 16-bit TIFF format. Then, raw data were converted using Imaris file converter software (Bitplane, Zurich, Switzerland) into ims. Images and videos were created using Imaris 9.3.0 software (Bitplane).

### Statistical analysis

GraphPad Prism software (Version 6.01) (GraphPad, San Diego, CA, USA) was used. Statistical significance of differences between mean values of two groups was determined using an unpaired two-tailed Student’s *t-*test. Differences across time points were analyzed using one-way ANOVA with post-hoc Tukey’s multiple comparisons tests. *P* < 0.05 was considered statistically significant.

### Study reporting

The study design, animal use and all experimental methods were conducted and reported in accordance with ARRIVE guidelines (https://arriveguidelines.org).

### Ethics approval and consent to participate

All animal studies were performed in compliance with the European guidelines, and the experimental protocol received approval from the Charles Darwin Ethics Committee for animal experimentation (Paris, France) (agreement APAFIS#23442-2019122316202171 v4).

## Results

### Lipopolysaccharide intranasal instillations induce pulmonary tertiary lymphoid structures formation

**Figure 1:**
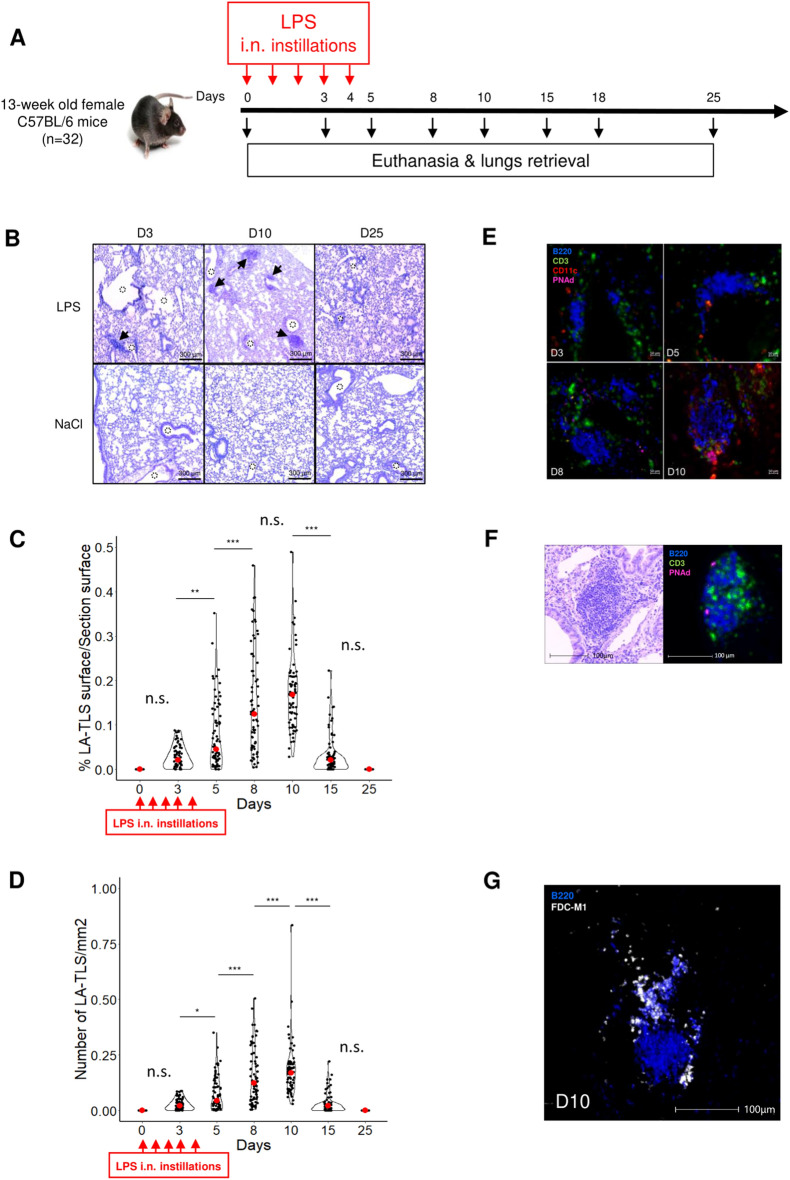
Transient induction of TLS formation following intranasal LPS instillation. (**A**) Mice were intranasally instilled daily from day 0 to day 4, and lungs were retrieved at days 0 (n = 4), 3 (n = 5), 5 (n = 5), 8 (n = 5), 10 (n = 5), 15 (n = 5) and 25 (n = 3) following LPS instillation (10 µg/mouse/day). Lungs were formalin-fixed and paraffin-embedded (FFPE), and sections were analyzed by hematoxylin and eosin (H&E) counterstaining and immunofluorescence staining. (**B**) H&E counterstaining at days 3, 10 and 25. Black arrowheads show lymphoid aggregates, and empty dotted circles show bronchi and/or alveoli lumen. (**C**) Violin plot showing lymphoid aggregate/TLS (LA-TLS) density as calculated using HALO software (see “Methods”). Each dot represents the LA-TLS density on one tissue section. Fifteen tissue sections per mouse were analyzed. Red dots represent the median values. (**D**) Violin plot showing the number of LA-TLS per tissue section as calculated using HALO software (see methods). Each dot represents the number of LA-TLS on one tissue section. Fifteen tissue sections per mouse were analyzed. Red dots represent the median values. (**E**) Immunofluorescence staining at days 3, 5, 8 and 10 shows B220^+^ B-cell aggregates, CD3^+^ T cells, CD11c^+^ myeloid cells (red), and PNAd^+^ HEV (day 8 and day 10). (**F**) H&E counterstaining (left panel) and immunofluorescence staining (right panel) of serial cuts of LPS-treated mouse lung at day 10. The right panel shows the B220^+^ B-cell area (blue), CD3^+^ T cells (green) and PNAd^+^ high endothelial venules (HEV, pink). (**G**) Immunofluorescence staining of a tissue section adjacent to that displayed in F (serial cuts), showing the presence of germinal centers with FDC-M1^+^ follicular dendritic cells (white) in the B-cell area. (**C**) and (**D**) were analyzed for statistical significance by one-way ANOVA with Tukey’s multiple comparison tests. It should be noticed that mice at day 3 have received a smaller cumulative dose of LPS than mice at all other days. *n.s.* not significant, *P < 0.05, ** *P* < 0.01, ***P < 0.001.

First, bronchus-associated TLS were induced in the pulmonary tissue of mice receiving lipopolysaccharide (LPS). Adult female mice received an intranasal injection daily for 5 days. Lung removal was performed on days 0, 3, 5, 8, 10, 15, 18 and 25 following the first injection of LPS. The experimental design is shown Fig. 1A. We observed the presence of a few small lymphoid aggregates in the close vicinity of the bronchus and blood vessels at day 3 by hematoxylin and eosin (H&E) counterstaining (Fig. 1B). The density and absolute number of aggregates increased thereafter and peaked at day 10 (Fig. 1C,D), with many aggregates evidenced at that time (Fig. 1B). Aggregates were no longer observed at day 25 (Fig. 1B–D). Statistical significance of the kinetics for day pairs is shown Fig. 1C,D.

At days 3 and 5 (i.e., during treatment and the day after the last LPS instillation), aggregates were mostly composed of B220^+^ B cells, with the presence of a few CD3^+^ T cells and CD11c^+^ myeloid cells in close proximity. No PNAd^+^ high endothelial venules (HEV) were detected at these time points (Fig. 1E). In parallel to the increase in size of lymphoid aggregates over time, PNAd^+^ HEV became detectable at day 8 (Fig. 1E). At day 10, these aggregates were composed of B220^+^ B cells, with the presence of CD3^+^ T cells and PNAd^+^ HEV, as detected by multiplexed immunofluorescence labeling of mouse lung sections. A representative lymphoid aggregate stained by H&E (left panel) and immunofluorescence (right panel) is shown Fig. 1F. B cell areas contained germinal centers displaying FDC-M1^+^ follicular dendritic cells (FDC). Figure 1G shows B220 and FDC-M1 staining at day 10 of a tissue section adjacent to the one in Fig. 1E. Thus, most of these aggregates were mature TLS. Control mice receiving NaCl intranasal instillations did not show any lymphoid aggregates in the lungs (Supplementary Fig. S1). In these mice, B220^+^ B cells and CD3^+^ T cells were scattered in the lung parenchyma. PNAd^+^ HEV and lymphoid aggregates were detected only in LPS-treated animals.

### 6-hydroxydopamine-mediated selective depletion of sympathetic peripheral fibers provokes a significant reduction in pulmonary alveolar space

**Figure 2:**
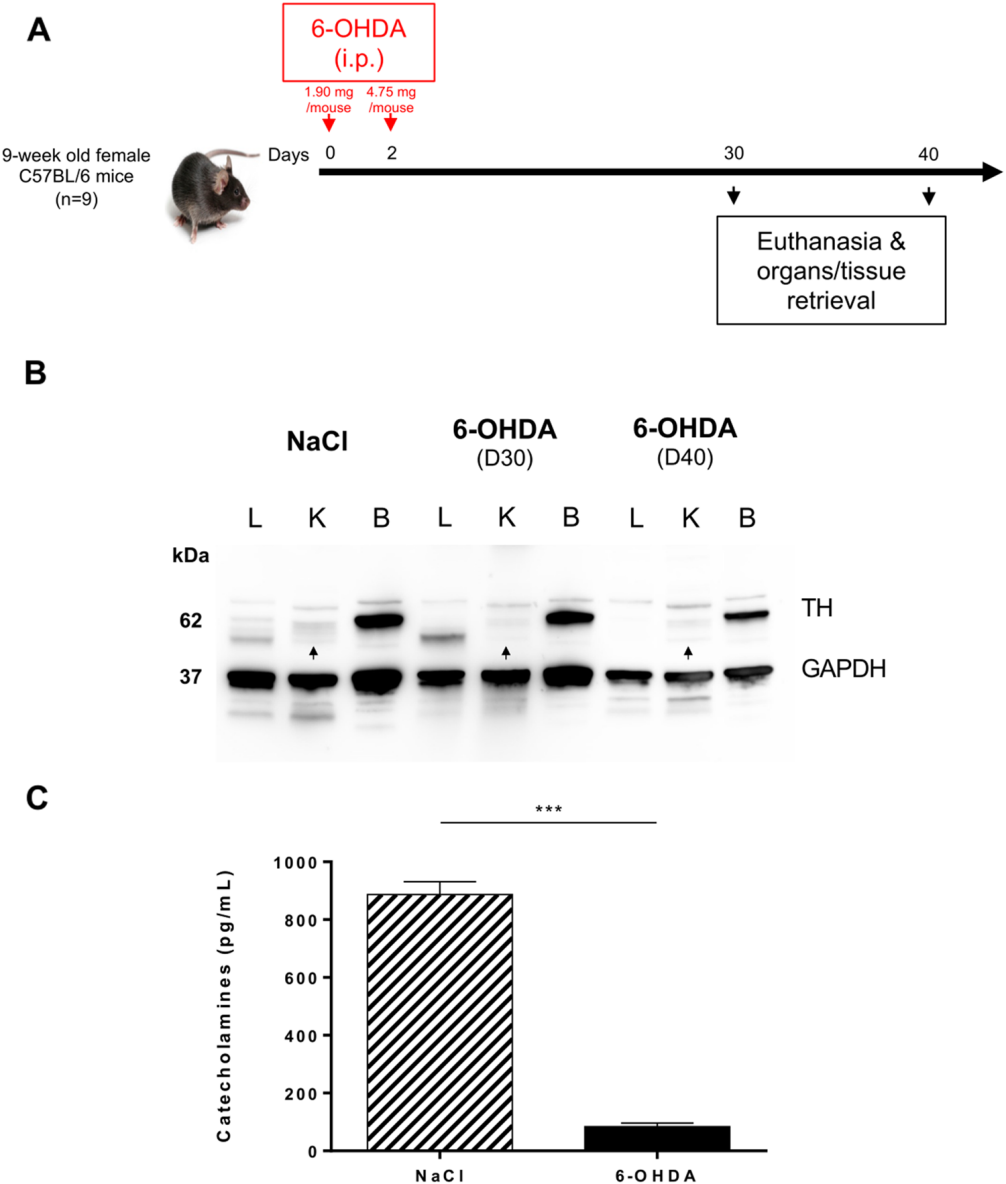
Depletion of sympathetic peripheral fibers following injection of 6-hydroxydopamine (6-OHDA). (**A**) Mice received intraperitoneal injections of 6-hydroxydopamine (6-OHDA) or NaCl (n = 9 for each group) at day 0 and day 2 (1.90 and 4.75 mg 6-OHDA/mouse, respectively). The lungs, kidney and brain were then retrieved at day 30 and day 40. (**B**) Western blot detection of TH and glyceraldehyde-3-phosphate dehydrogenase (GAPDH) present in tissue extracts from the lung (L), kidney (K) and brain (B) of 6-OHDA- and NaCl-treated animals at day 30 and day 40. Original blot and molecular size marker are shown in Supplementary Fig. S3. (**C**) Quantification of catecholamine concentration in the plasma of 6-OHDA- and NaCl-treated animals at day 40. Histogram bars represent the S.E.M. of duplicate ELISA values from 6-OHDA- (n = 4) and NaCl-treated (n = 9) animals. Data were analyzed for statistical significance by unpaired two-tailed Student’s *t*-test. ***P < 0.001.

We explored the impact of the depletion of sympathetic peripheral fibers on LPS-induced lymphoid aggregates and TLS. For that purpose, we used 6-hydroxydopamine (6-OHDA), a neurotoxin that selectively destroys any cell expressing tyrosine hydroxylase (TH), including sympathetic peripheral fibers^16^. First, the impact of two subsequent intraperitoneal (i.p.) injections of 6-OHDA (day 0 and day 2) on nerve fibers of naive C57BL/6 mice was examined. Figure 2A shows the experimental design. TH was detected neither at day 30 nor at day 40 in 6-OHDA-treated mouse ears in comparison to control mice receiving NaCl (Supplementary Fig. S2).

**Figure 3:**
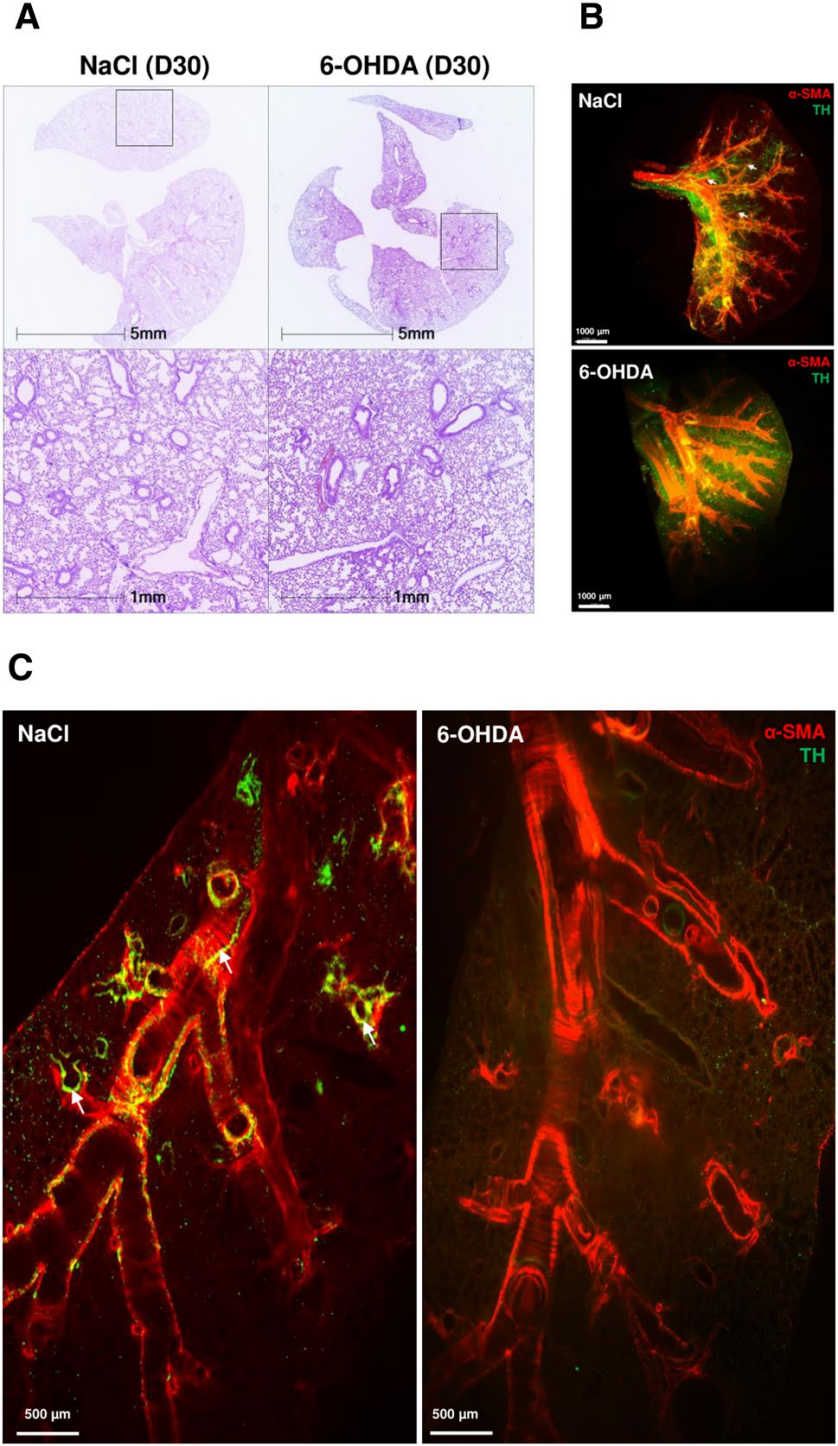
6-OHDA treatment induces alveolar space reduction and TH^+^ fiber depletion. (**A**) H&E counterstaining of FFPE lung sections of NaCl (left panel) and 6-OHDA (right panel) -treated animals at day 30. Areas in the black squares in the upper panels are enlarged in the corresponding lower panel. (**B**) Snapshots of 3D views of light-sheet fluorescence microscopy (LSFM) of 6-OHDA- and NaCl-treated mouse lungs at day 40 showing TH (green) and α-SMA (red) staining. White arrows illustrate the presence of TH^+^ sympathetic fibers in NaCl-treated mouse data. (**C**) 2D staining of 6-OHDA- and NaCl-treated mouse lungs at day 40 showing TH (green) and α-SMA (red) staining. White arrows illustrate the presence of TH^+^ sympathetic fibers in NaCl-treated mice.

To assess the drug efficacy in ablating sympathetic nerve fibers in various organs of treated mice, western blots revealing the presence of TH, that exhibits a 58–60 kDa molecular weight, were performed on lung, kidney and brain extracts (Fig. 2B and Supplementary Fig. S3). TH levels in the brain were not modified following 6-OHDA injections, confirming that the drug does not cross the blood–brain barrier (BBB)^16^. Kidney extracts from NaCl-treated mice showed a weaker signal compared to brain extracts, and that signal was barely detected in kidneys of 6-OHDA-treated mice compared to NaCl-treated mice, confirming the well-known systemic neurotoxicity of 6-OHDA (Fig. 2B, black arrowheads). TH western blot signal could not be assessed in lung tissues. Only two very faint signals were detected in the 58–60 kDa area in NaCl-treated animals, making it impossible to define which signal corresponded to TH. However, 2D and 3D imaging showed that no TH^+^ fibers could be detected in the lungs of 6-OHDA-treated mice (Fig. 3B, lower panel, and Fig. 3C, right panel) (see also Supplementary Videos S1 and S2). A considerable drop in catecholamine levels (adrenaline, noradrenaline and dopamine) in the plasma of 6-OHDA-treated mice was also observed by ELISA (Fig. 2C), suggesting a massive and systemic destruction of sympathetic fibers in these animals, except in the brain due to the inability of 6-OHDA to cross the BBB.

We then assessed the pulmonary structure of treated mice on lung tissue sections at day 30. A reduction in the alveolar surface was detected in the pulmonary parenchyma of 6-OHDA-treated mice compared to control mice (Fig. 3A). When gross macroscopic appearance and behaviors were examined, no respiratory signs (difficulty in breathing, sniffling, sneezing) were recorded in treated animals, although no detailed metrics of respiratory functions (such as treadmill exercise tolerance testing) were performed. Remarkably, neither significant difference in weight nor decreased activity or ruffled fur were observed at day 30 post-6-OHDA injections between the animals of the two groups (6-OHDA-treated and NaCl-treated). TH^+^ sympathetic innervation of PFA-fixed whole lungs in 6-OHDA- and NaCl-treated animals was next visualized at day 40 by 2D and 3D imaging. The α-SMA^+^ network was detected in both groups (Fig. 3B,C). These smooth muscular cells were surrounded by TH^+^ sympathetic fibers in control mice (Fig. 3B, upper panel, and C, left panel). In contrast, no fibers but only green scattered dots could be detected in the lungs of 6-OHDA-treated mice (Fig. 3B, lower panel, and C, right panel) (see also Supplementary Videos S1 and S2).

### Depletion of sympathetic fibers leads to a decrease in LPS-induced lymphoid aggregates and TLS formation in lung parenchyma

**Figure 4:**
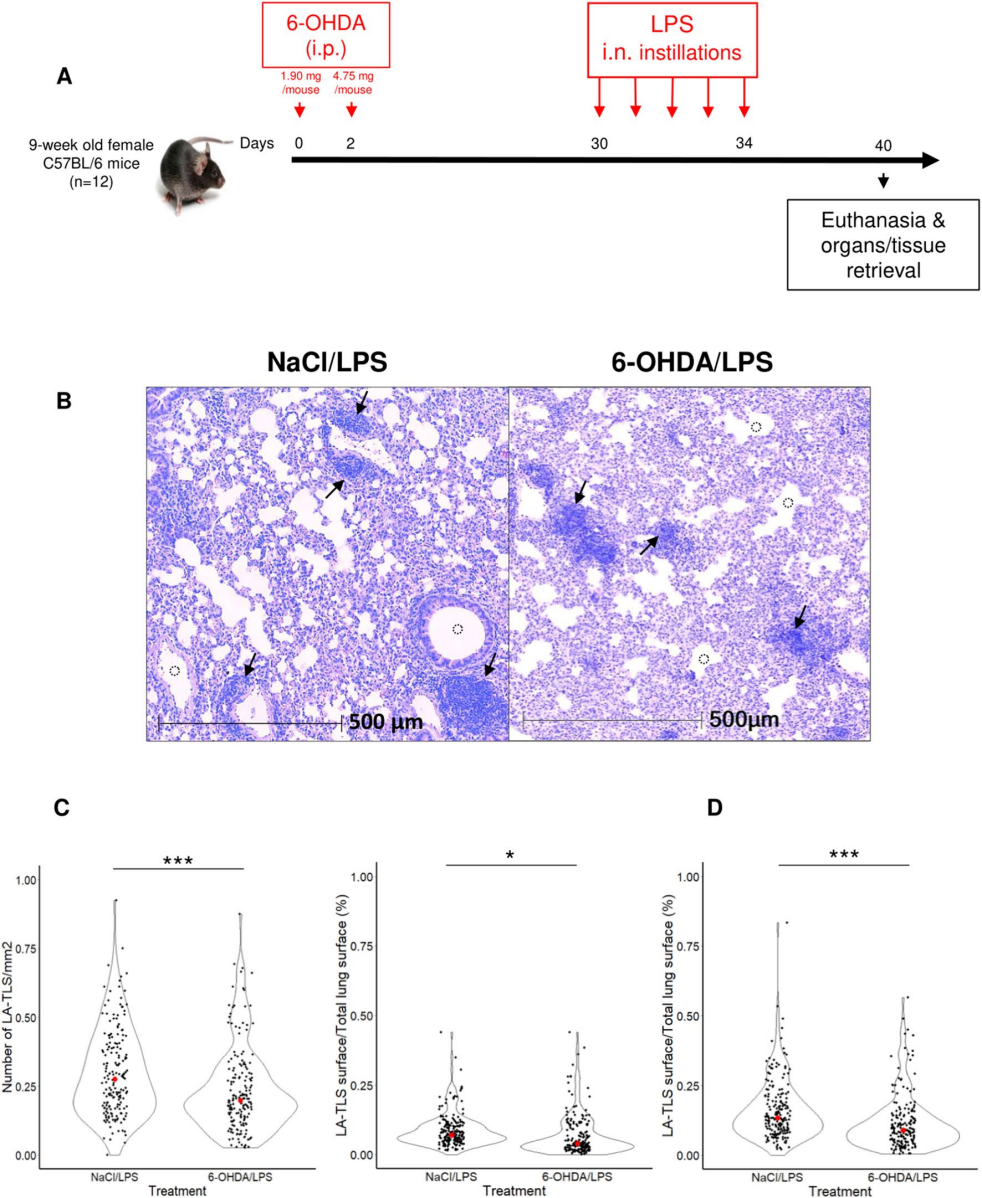
Decrease in the formation of lung lymphoid aggregates following 6-OHDA depletion of sympathetic fibers. (**A**) Mice received intraperitoneal (i.p.) injections of 6-OHDA (n = 12) at day 0 and day 2 followed by intranasal (i.n.) daily instillations of LPS (10 µg/mouse/day) from day 30 to day 34. Control mice (n = 13) received NaCl instead of 6-OHDA. As day 30 was selected as the starting time for LPS instillations, mice were euthanized and lungs were retrieved at day 40, i.e., ten days later, when TLS density and number were maximal (Fig. 1C,D), and TH^+^ fibers were still undetectable (Fig. 2C and Supplementary Fig. S2). FFPE lung sections were analyzed by H&E counterstaining and immunofluorescence staining. (**B**) H&E counterstaining of mouse lung sections (day 40) following 6-OHDA/LPS or NaCl/LPS treatment. Black arrowheads show lymphoid aggregates, and empty dotted circles show bronchi and/or alveoli lumen. (**C**) Violin plots showing lymphoid aggregates, most of them being TLS (indicated as LA-TLS) number (left panel) and LA-TLS density (right panel), calculated using HALO software as described in the methods. (**D**) Violin plot showing LA-TLS density, with consideration of alveolar space in the calculation, using HALO software as described in the methods. Each dot represents the value for one tissue section. Fifteen tissue sections per mouse were analyzed. Red dots represent the median value for each group. (**C**) and (**D**) were analyzed for statistical significance by unpaired two-tailed Student’s *t*-test. *P = 0.0140, ***P < 0.001.

We assessed LPS-induced TLS formation in the lungs of mice lacking sympathetic nerve fibers. 6-OHDA- and NaCl-treated mice were given LPS intranasal instillations (Fig. 4A). Day 30 was selected as the starting time for LPS instillations, and lungs were retrieved at day 40, i.e., 10 days later, when TLS density and number were maximal (Fig. 1C,D) and TH^+^ fibers were still undetectable (Fig. 2B). Sympathetic denervation was verified by whole-mount TH staining of 6-OHDA-treated mouse ears (Supplementary Fig. S2). H&E counterstaining of paraffin-embedded lung sections showed the presence of lymphoid aggregates in both 6-OHDA- and NaCl-treated mouse lungs (Fig. 4B). The number of lymphoid aggregates observed per lung section, most of which were TLS (indicated as LA-TLS), was significantly lower in 6-OHDA-treated mice than in NaCl-treated controls (*P* = 0.0006, Fig. 4C, left panel). A decrease in the density of LA-TLS was also observed in 6-OHDA-treated mice compared to control animals (*P* = 0.0140, Fig. 4C, right panel). As previously observed **(**Fig. 3A**)**, the lung parenchyma of 6-OHDA-treated mice contained fewer and smaller alveoli than that of control mice (Fig. 4B). Thus, we examined whether this change in lung parenchyma architecture impacts LA-TLS density assessment. Quantification of the ratio of alveolar surface to total lung surface also showed a decrease following 6-OHDA treatment (*P* < 0.0001) (Supplementary Fig. S4). Quantification of LA-TLS density in the lung tissue excluding the alveolar surface confirmed the difference between the two groups (*P* < 0.0001) (Fig. 4D). Assessment of lung inflammatory status following LPS treatment by real-time quantitative PCR showed that the day after the last instillation (i.e. day 35), expression levels of genes encoding TNF-α, IL-1β and IFN-γ inflammation cytokines were increased in the lungs of LPS-treated mice, whether or not they had received 6-OHDA prior to LPS treatment (Supplementary Figs. S5 and S6, and Supplementary Table S3). In line with the local inflammation triggered by 6-OHDA treatment in some experimental settings, higher mean expression levels were even found in the lungs of denervated mice, in which alveolar surface reduction was observed at day 40 in the lungs of these animals (Fig. 4B).

In parallel, immune cell infiltration in the lungs of NaCl/LPS- and 6-OHDA/LPS-treated mice was also examined the day after the last instillation (day 35) by flow cytometry. It showed no difference between groups in the proportions of CD45^+^ pulmonary cells (Supplementary Fig. S7A). Among these cells, changes were observed neither in the proportion of CD19^+^ B220^+^ B cells (Supplementary Fig. S7B) nor in the proportion of CD3^+^ T cells (Supplementary Fig. S7C).

**Figure 5:**
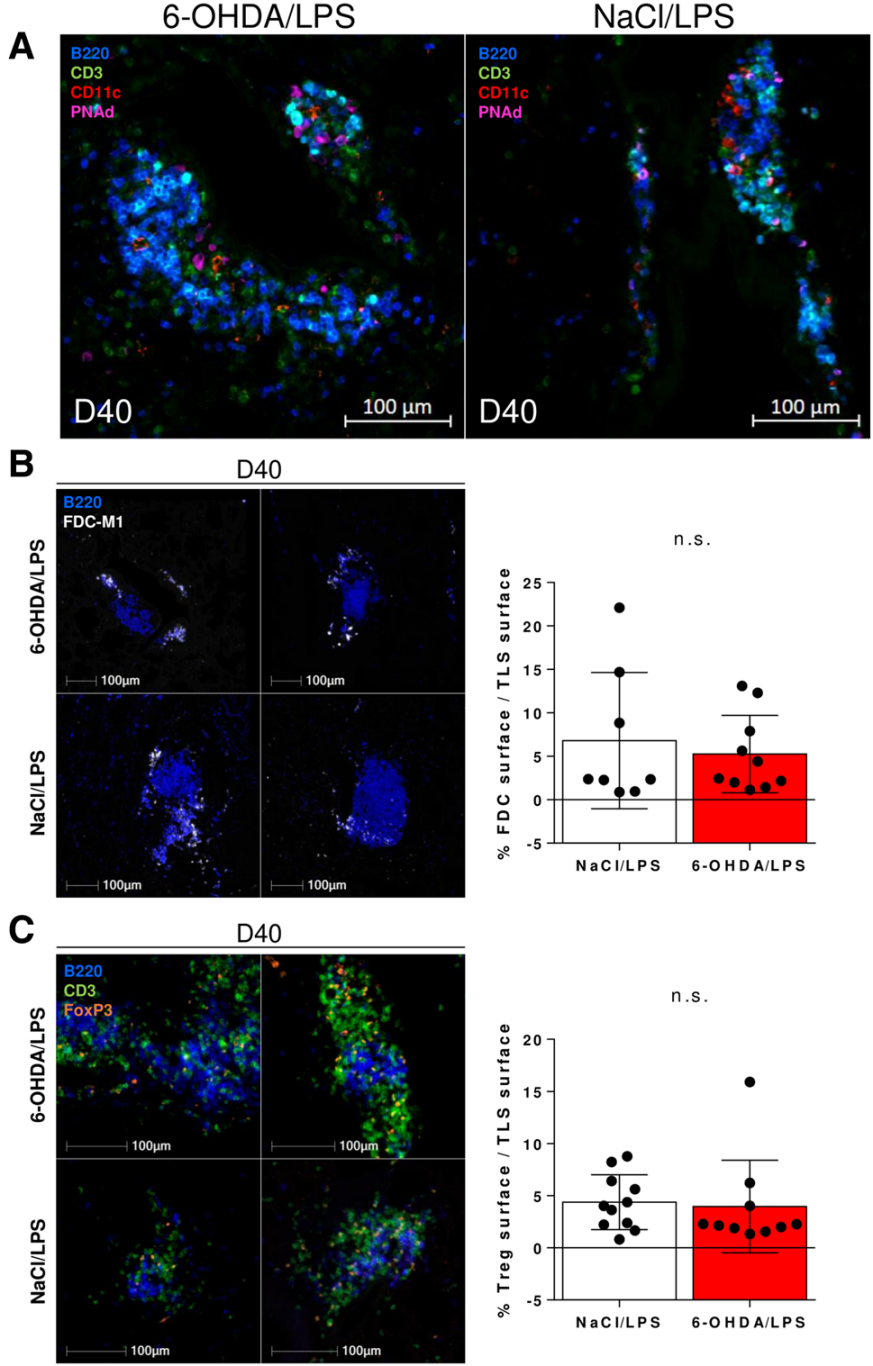
Most of the lymphoid aggregates observed in 6-OHDA-treated mice are mature TLS. (**A**) Immunofluorescence staining of lung sections from 6-OHDA/LPS (left panel) and NaCl/LPS (right panel) -treated mice showing a TLS containing B220^+^ B cells (blue), CD3^+^ T cells (green), CD11c^+^ myeloid cells (red) and PNAd^+^ HEV (pink). (**B**) Immunofluorescence staining of lung sections from 6-OHDA/LPS (upper panels) and NaCl/LPS (lower panels) -treated mice showing the presence of FDC-M1^+^ FDC (white) in the B-cell zone of TLS. (**C**) Immunofluorescence staining of lung sections from 6-OHDA/LPS (upper panels) and NaCl/LPS (lower panels) -treated mice showing B220^+^ B cells (blue), CD3^+^ T cells (green) and FoxP3^+^ regulatory T cells (orange). (**B**,**C**) Left and right panels show the staining of different TLS. Histogram graphs show the mean values of FDC (**B**) or Treg (**C**) surface on TLS surface, within TLS observed in 6-OHDA/LPS- or NaCl/LPS-treated mice. Each dot represents the value for one observed TLS. Difference in the means of each group was analyzed using unpaired two-tailed Student’s *t*-test. *n.s.* not significant [*P* = 0.6022 (**B**) or *P* = 0.7954 (**C**)].

Multiplexed immunofluorescence staining of adjacent sections confirmed that most of the lymphoid aggregates were TLS (containing B220^+^ B cells, CD3^+^ T cells, CD11c^+^ myeloid cells and PNAd^+^ high endothelial venules) (Fig. 5A). The maturation stage of induced TLS was then assessed 10 days after the beginning of LPS instillations (day 40). FDC-M1^+^ FDC were observed within the B-cell areas in the lungs of both 6-OHDA- and NaCl-treated mice, indicating the presence of mature TLS (Fig. 5B). TLS infiltration by FoxP3^+^ Tregs in 6-OHDA- *vs* NaCl-treated mice was similar at day 40 (Fig. 5C).

### Pulmonary naive B cell levels after LPS treatment are decreased following sympathetic fiber depletion

**Figure 6:**
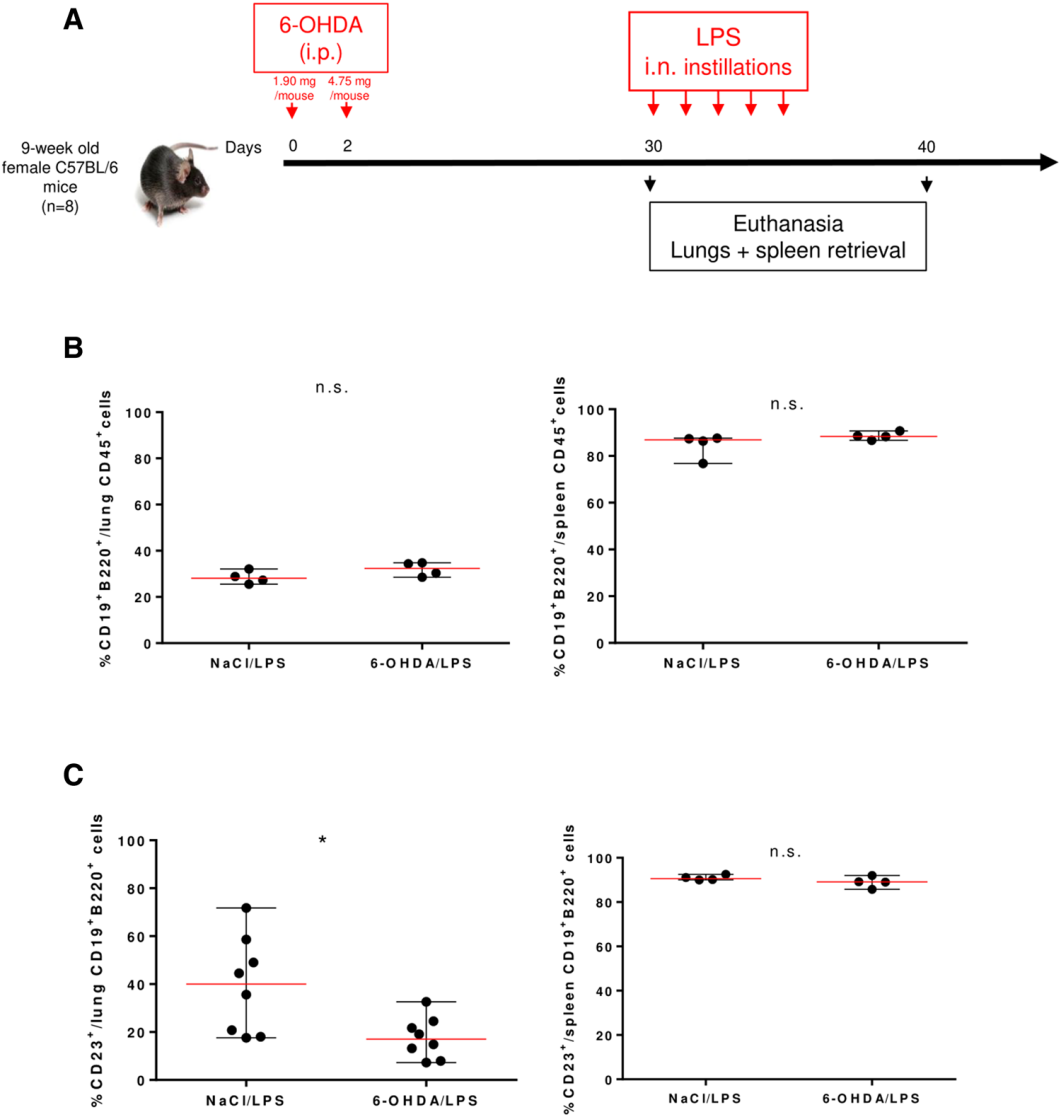
Impact of sympathetic denervation on immune cell subsets. (**A**) Mice received intraperitoneal (i.p.) injections of 6-OHDA (n = 12) at day 0 and day 2 followed by intranasal (i.n.) daily instillations of LPS (10 µg/mouse/day) from day 30 to day 34. Control mice (n = 12) received NaCl instead of 6-OHDA. Mice were euthanized, and the lungs (n = 8) and spleen (n = 4) were retrieved at day 30 and day 40. (**B**) Proportions of CD19^+^B220^+^ B cells among CD45^+^ pulmonary (left panel) and splenic (right panel) leukocytes at day 40. (**C**) Proportions of CD23^+^ naive B cells among pulmonary (left panel) and splenic (right panel) CD19^+^B220^+^ B cells. (**B**,**C**) were analyzed for statistical significance by unpaired two-tailed Student’s *t*-test. *n.s.* not significant, **P = 0.0015.

We assessed whether sympathetic fiber depletion impacts immune cell subsets in the lungs and spleen prior to LPS treatment, when no LA-TLS are detected in the lungs, and after treatment. Animals were i.p. injected with 6-OHDA or NaCl at day 0 and day 2 and intranasally treated with LPS starting at day 30. Spleens and lungs were collected at days 30 and 40 (Fig. 6A), and recovered immune cells were analyzed by flow cytometry. Gating strategies are shown in Supplementary Figs. S8 and S9. 6-OHDA treatment did not affect CD45^+^ leukocyte proportions among live cells in the spleen and in the lungs at day 40 after receiving LPS. Among them, the proportions of CD19^+^B220^+^ B cells (Fig. 6B and Supplementary Table S4), CD3^+^ T cells and IA/IE^+^ CD19^-^ cells (Supplementary Table S4) were not altered in 6-OHDA-treated mice compared to NaCl-treated animals. However, at day 40 following LPS treatment, a significant difference in the proportion of CD23^+^ naive cells among CD19^+^ B220^+^ B cells was observed between 6-OHDA- and NaCl-treated mouse lungs (18.12% *vs* 43.50%, *P* = 0.0015, Fig. 6C left panel and Supplementary Table S4), while no difference was observed at day 30 (40.95% *vs* 33.48%, *P* = 0.3086, data not shown). At day 33, i.e*.* after 3/5 intranasal instillations of LPS, a decrease in CD23^+^ B cell proportion was already observed in the lungs (19.93% *vs* 48.30%, *P* = 0.0111) (Supplementary Fig. S10). No drop in naive B cells was observed in the spleen (Fig. 6C right panel and Supplementary Table S4). Finally, no difference in FoxP3^+^ Treg proportions among lung CD4^+^ helper T cells was observed between the two groups following LPS instillations (Supplementary Table S4), as already shown by immunofluorescence on tissue sections (Fig. 5C). 6-OHDA treatment also had no impact on the expression of ICOS and PD-1 immune checkpoints by helper T cells in the spleen or lungs of treated mice (Supplementary Table S4).

## Discussion

This work shows that TLS form transiently in mouse lungs inflamed by LPS and that their formation is hampered by sympathetic nerve fiber depletion. TLS progressive disappearance when inflammation resolves corroborates previous findings using the same treatment^17^. No TLS were detected in the lungs three weeks after the last instillation. In contrast, mice pretreated with LPS as neonates exhibited TLS at that time. The authors suggested that there is a developmental window after birth when TLS form more easily in the lungs upon inflammatory stimuli. This would make these mice more prone to form TLS under inflammatory conditions as adults. However, in this study, the presence of TLS was not examined before the third week post-instillation in adult mice that did not receive LPS as neonates.

We also show that TLS formation following acute inflammation in mouse lungs is hampered in the absence of sympathetic innervation. Remarkably, no difference in the size of lymphoid aggregates was visible on tissue sections between control and denervated mice. Our findings are in favor of a reduced efficiency in TLS formation due to absence of local sympathetic nerve fibers. The chemical ablation of local nerve fibers could lead to a decrease in the production of molecules important for the early steps of TLS formation that require a crosstalk between lymphoid tissue inducer immune cells and stromal lymphoid tissue organizer cells within lung tissue. It would limit the intensity of the signaling necessary to TLS organization and, hence, reduce their numbers, but without affecting those under construction*.* Notably, TLS cellular composition and maturation stage were comparable between both groups of animals, with the presence of HEV and comparable densities of Treg and FDC, the latter advocating for the existence of germinal centers.

Smaller alveoli were observed following 6-OHDA treatment. Whether the lower number and density of TLS are a consequence of a reduced inhalation of LPS due to lung impairment is unlikely. Indeed, real-time qPCR and analysis of immune cell infiltration in the lungs of NaCl/LPS- or 6-OHDA/LPS-treated mice invalidated the assumption of an indirect impact of sympathetic fiber depletion through a lower LPS stimulation of inflammation.

Several signaling pathways act redundantly to generate TLS^18^. Thus, compensatory pathways could rely on other nerve fibers to induce TLS formation in the absence of sympathetic nervous system involvement. In a murine model of dextran sulfate sodium-induced colitis, no change in TLS formation was observed after surgical sympathectomy in the colon^19^. However, it was significantly reduced in vagotomized mice, along with a decrease in B-cell-attracting chemokines. In another study, 6-OHDA-mediated denervation of sympathetic nerve fibers induced the collapse of arterial plaque-associated lymphoid structures^13^. All these data suggest that the crosstalk between TLS and the nervous system is context and perhaps tissue dependent.

The denervation procedure may also be a major parameter to consider. Mice undergoing surgical vagotomy in the proximal colon displayed decreased TLS in this area but not in the distal colon, where intact parasympathetic innervation could still be observed^19^. In contrast, in our setting, the administration of 6-OHDA led to a systemic depletion of sympathetic fibers, as shown by the massive drop in plasma catecholamines accompanied by a decrease in TLS formation.

Moreover, we observed no impact of i.p. 6-OHDA treatment on the levels of pulmonary or splenic CD45^+^ leukocytes and no change in the proportions of B and T lymphocytes among these cells. These findings suggest that the removal of sympathetic fibers does not alter the recruitment of immune cells from the blood stream. This idea is supported by the detection of an immune infiltrate with scattered B and T cells observed in addition to TLS in the pulmonary tissue of denervated mice. However, although no change in total B cell levels was detected, we observed a decreased proportion of lung naive B cells following LPS treatment in mice treated with 6-OHDA. It has been shown that B cells express β2-adrenergic^20^ and dopamine^7^ receptors, and that Tfh release stored dopamine to facilitate Tfh-B cell interactions and accelerate germinal center productive synapses^21^. One can hypothesize that the drastic decrease in catecholamines due to 6-OHDA treatment could therefore affect the formation and function of TLS in denervated mice in response to LPS stimulation, which triggers IgM production by B cells^22^. It is known that sympathetic fibers act on B cell compartments and antibody response^23,24^. In particular, IgM antibody response to bovine serum albumin and B cell number have been found to be significantly decreased in mice producing only minute amounts of norepinephrine (noradrenaline)—a major neurotransmitter produced by sympathetic nerve fibers—in spleen and lymph nodes^23^. Thus, these data show that secondary lymphoid organs are sensitive to the presence of neurotransmitters produced by sympathetic nerve fibers. It suggests that the same crosstalk may occur in tertiary lymphoid structures. In our setting, analysis of the antibody response to ovalbumin five days after intraperitoneal immunization resulted in a lower anti-ovalbumin IgM response in most of the 6-OHDA-treated mice compared to control mice, although this result is yet to be confirmed (data not shown). Of note, peribronchial LA neogenesis during bacterial inflammation has been shown to be sustained in the absence of B cells. In that setting, LA were mostly composed of T cells and HEV but did not display any germinal center^25^.

The role of the different nerve fibers with regard to HEV and TLS neogenesis and maintenance has yet to be elucidated, given the complexity of the peripheral nervous system and the variety of inflammatory models used. It has been recently shown that sensory nerve fiber ablation promotes HEV maturation, recruitment of immune cells and TLS organization in melanoma^24^. In contrast, ablation of motor fibers, in particular parasympathetic nerves, hinders TLS formation in the gut in the context of colitis^19^. This complexity is also underlined by the heterogeneous effects of the sympathetic nervous system depending on the nature of the adrenergic receptors involved (α or β) and their prior exposure to catecholamines^26^. All these data highlight the complexity of deciphering the mechanisms by which sympathetic nerve fibers support TLS neogenesis under inflammatory conditions.

### Supplementary Information

Supplementary Figures.

Supplementary Tables.

Supplementary Video 1.

Supplementary Video 2.

Supplementary Legends.

## Data Availability

The datasets used and/or analyzed during the current study are available from the corresponding author on reasonable request.

## Abbreviations

6-OHDA: 6-Hydroxydopamine
α-SMA: Alpha-smooth muscle actin
DCM: Dichloromethane
FFPE: Formalin-fixed paraffin embedded
HEV: High endothelial venule
iBALT: Inducible bronchus-associated lymphoid tissue
ICP: Immune checkpoint
iDISCO: Immunolabeling-enabled three-dimensional imaging of solvent-cleared organs
LA: Lymphoid aggregate
LPS: Lipopolysaccharide
LSFM: Light-sheet fluorescence microscopy
PFA: Paraformaldehyde
PNAd: Peripheral node addressin
TH: Tyrosine hydroxylase
TLS: Tertiary lymphoid structure
