# Supplementary material for: Absence of sympathetic innervation hampers the generation of tertiary lymphoid structures upon acute lung inflammation
Source: Sci Rep. 2024 May 23;14:11749. doi: 10.1038/s41598-024-62673-0 (PMC11116507; doi:10.1038/s41598-024-62673-0)
Supplement: Supplementary file 1 — Supplementary Figures. [file 41598_2024_62673_MOESM1_ESM.pptx]

## Slide 1
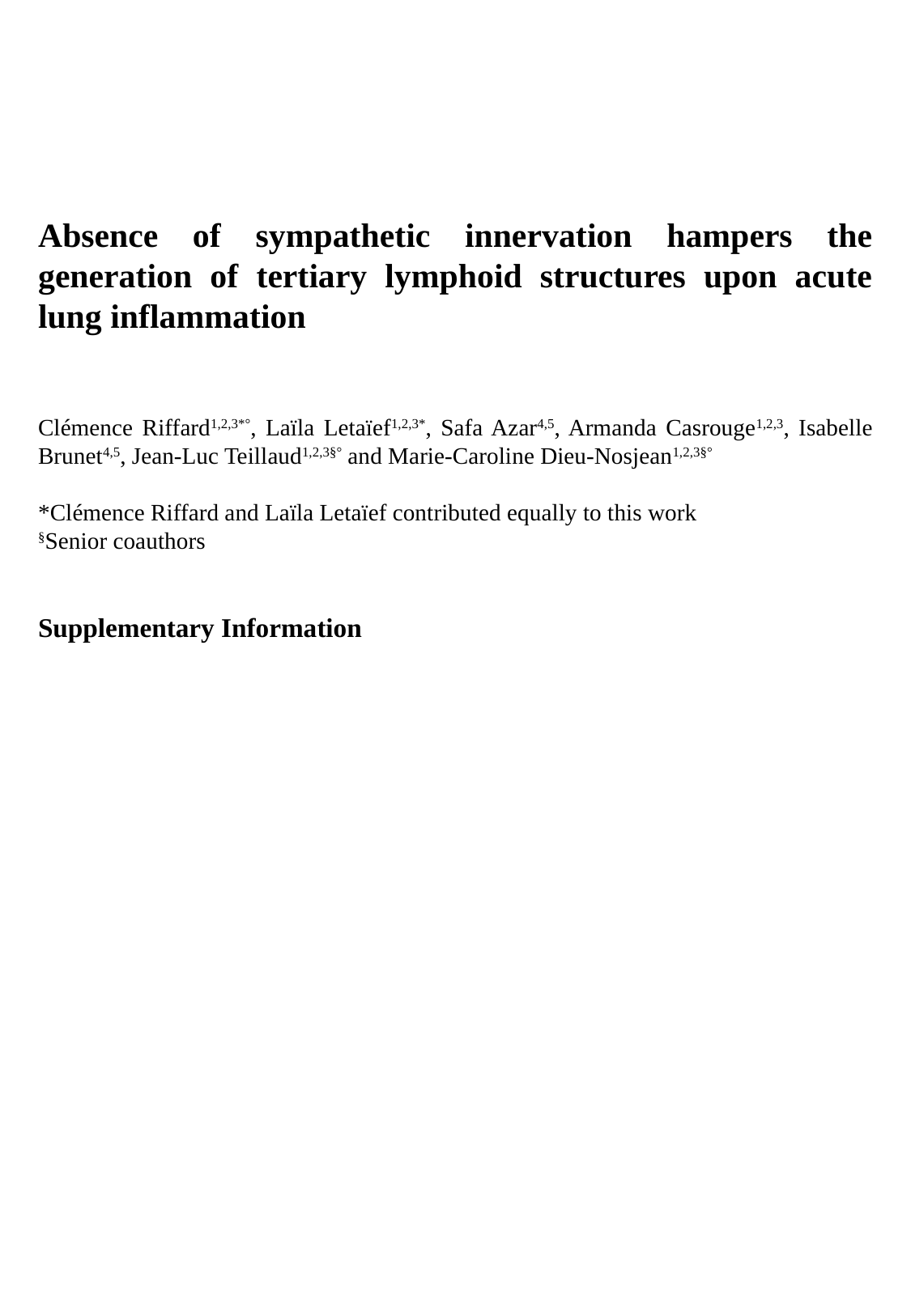

Absence of sympathetic innervation hampers the generation of tertiary lymphoid structures upon acute lung inflammation
Clémence Riffard1,2,3*°, Laïla Letaïef1,2,3*, Safa Azar4,5, Armanda Casrouge1,2,3, Isabelle Brunet4,5, Jean-Luc Teillaud1,2,3§° and Marie-Caroline Dieu-Nosjean1,2,3§°
*Clémence Riffard and Laïla Letaïef contributed equally to this work
§Senior coauthors
Supplementary Information

## Slide 2
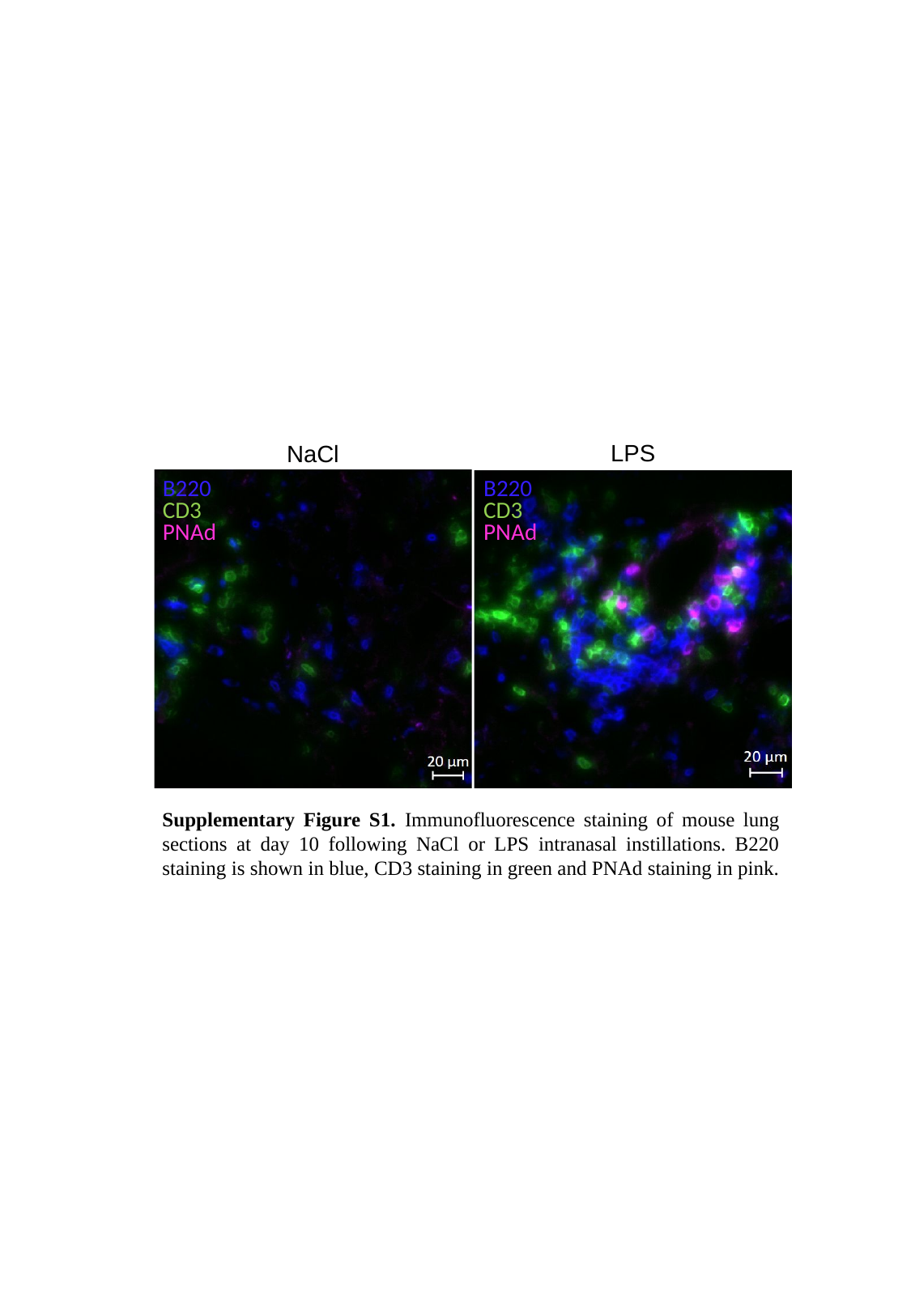

LPS
NaCl
Supplementary Figure S1. Immunofluorescence staining of mouse lung sections at day 10 following NaCl or LPS intranasal instillations. B220 staining is shown in blue, CD3 staining in green and PNAd staining in pink.
B220
CD3
PNAd
B220
CD3
PNAd
D10

## Slide 3
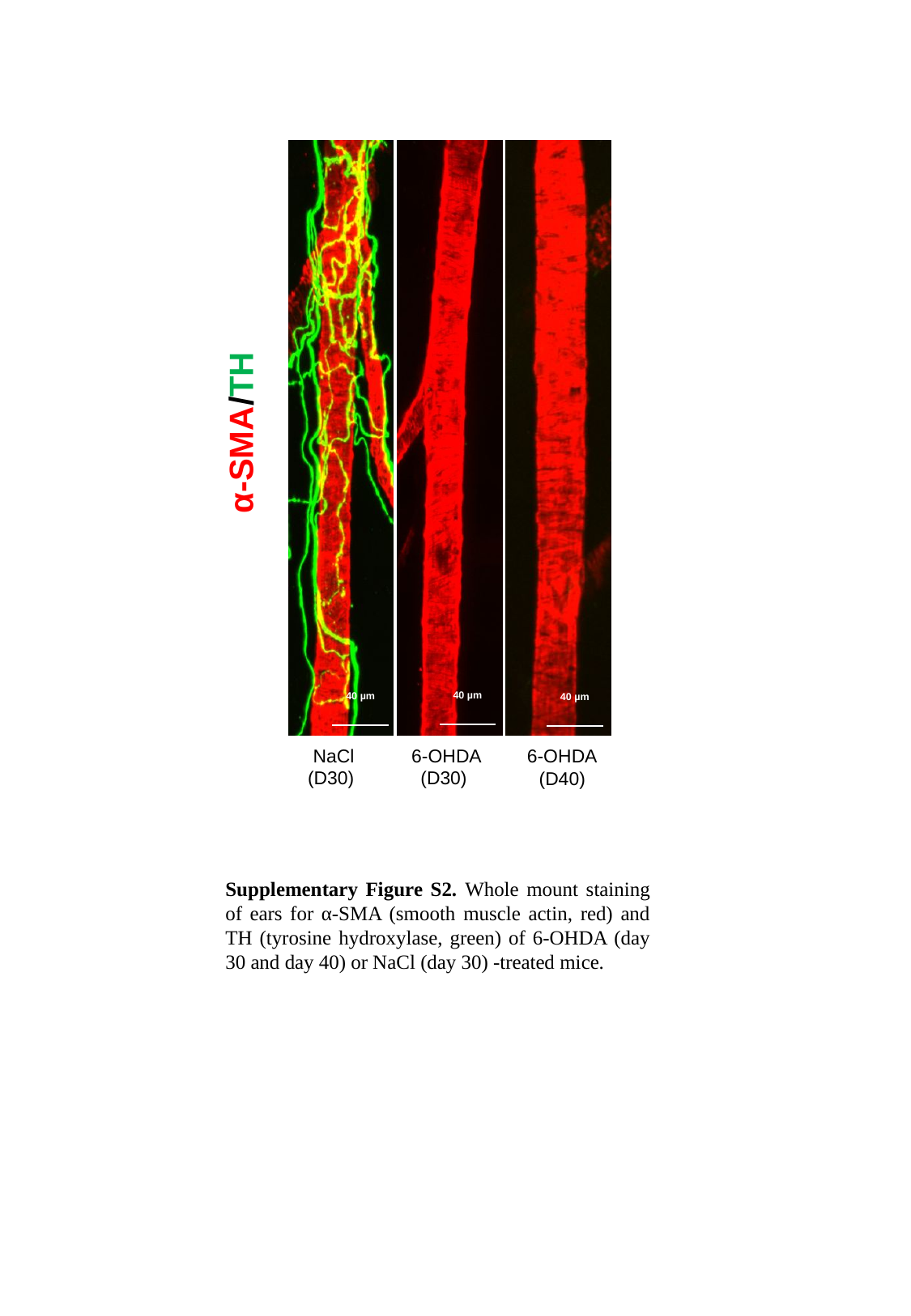

α-SMA/TH
40 µm
NaCl
(D30)
6-OHDA
(D30)
6-OHDA
(D40)
40 µm
40 µm
Supplementary Figure S2. Whole mount staining of ears for α-SMA (smooth muscle actin, red) and TH (tyrosine hydroxylase, green) of 6-OHDA (day 30 and day 40) or NaCl (day 30) -treated mice.

## Slide 4
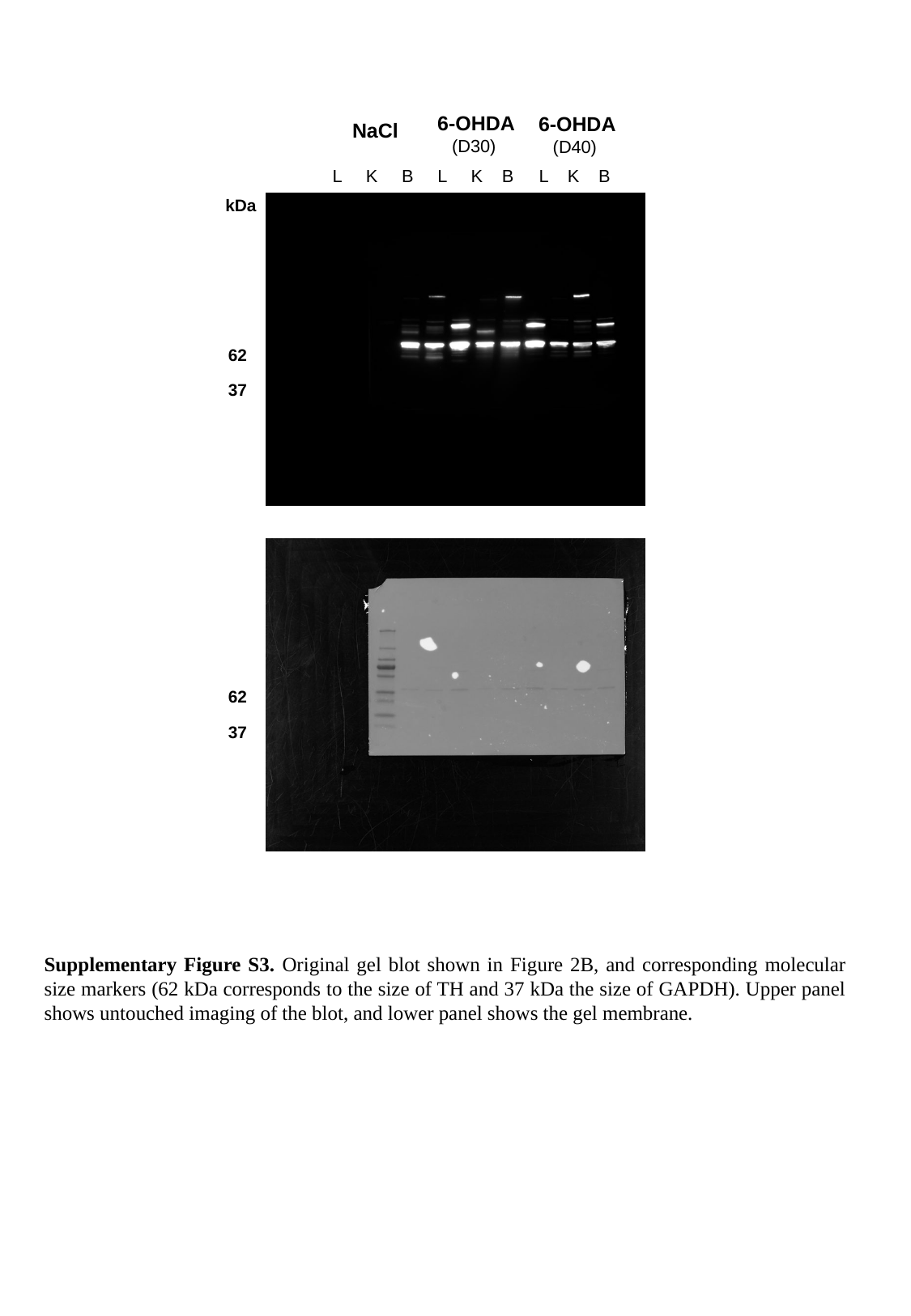

6-OHDA
(D30)
6-OHDA
(D40)
NaCl
L K B
 L K B
kDa
62
37
62
37
 L K B
Supplementary Figure S3. Original gel blot shown in Figure 2B, and corresponding molecular size markers (62 kDa corresponds to the size of TH and 37 kDa the size of GAPDH). Upper panel shows untouched imaging of the blot, and lower panel shows the gel membrane.

## Slide 5
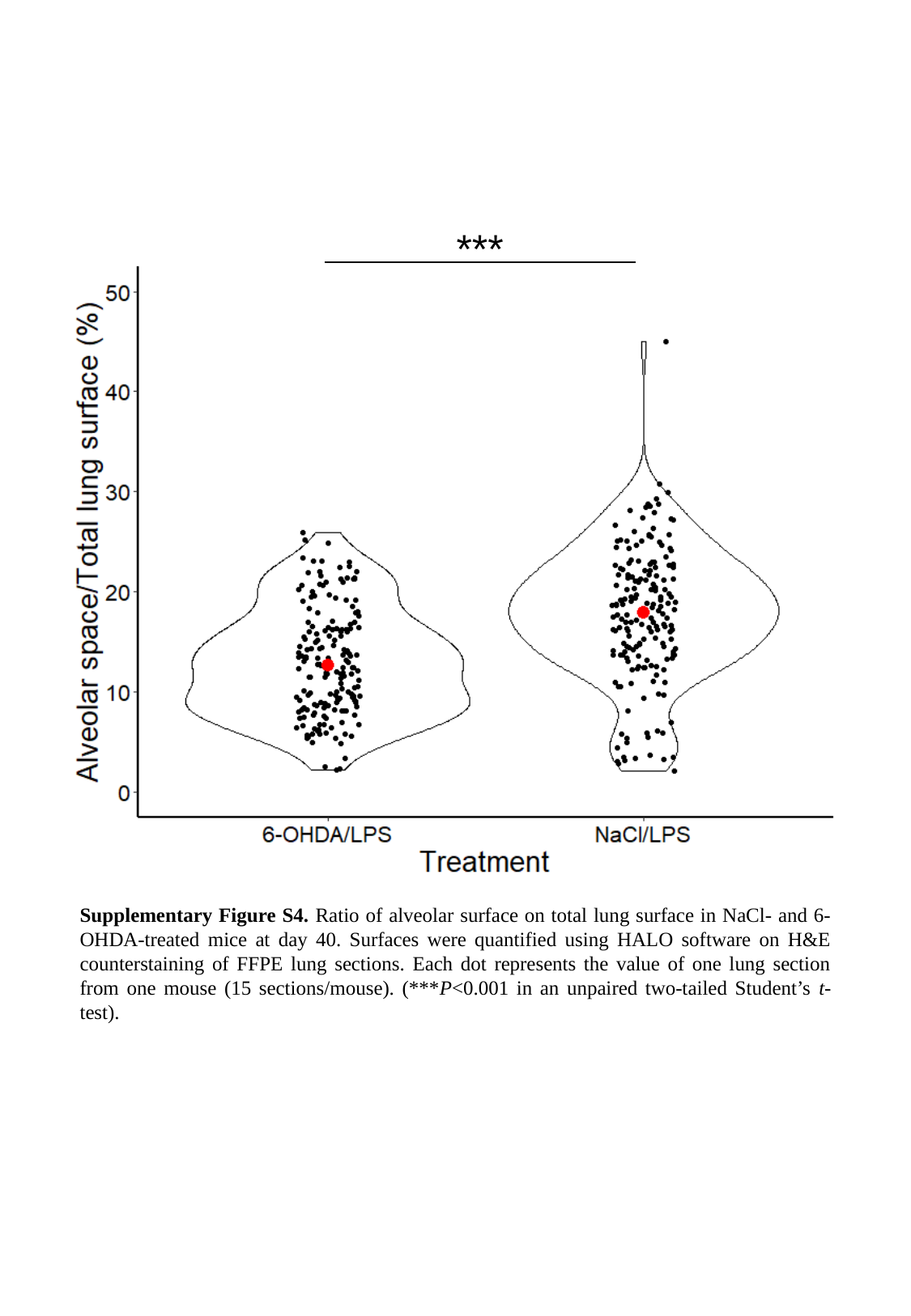

***
Supplementary Figure S4. Ratio of alveolar surface on total lung surface in NaCl- and 6-OHDA-treated mice at day 40. Surfaces were quantified using HALO software on H&E counterstaining of FFPE lung sections. Each dot represents the value of one lung section from one mouse (15 sections/mouse). (***P<0.001 in an unpaired two-tailed Student’s t-test).

## Slide 6
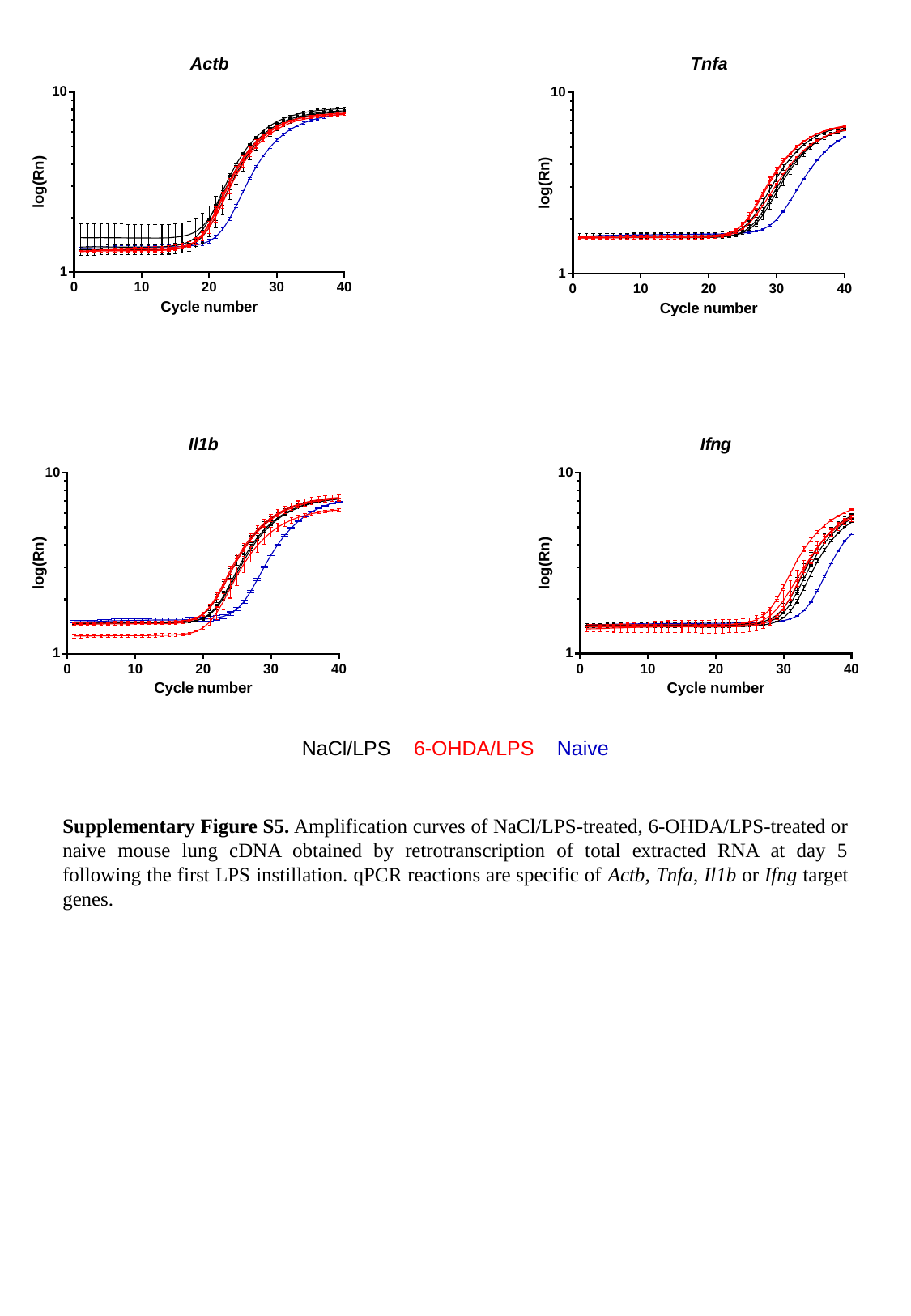

NaCl/LPS 6-OHDA/LPS Naive
Supplementary Figure S5. Amplification curves of NaCl/LPS-treated, 6-OHDA/LPS-treated or naive mouse lung cDNA obtained by retrotranscription of total extracted RNA at day 5 following the first LPS instillation. qPCR reactions are specific of Actb, Tnfa, Il1b or Ifng target genes.

## Slide 7
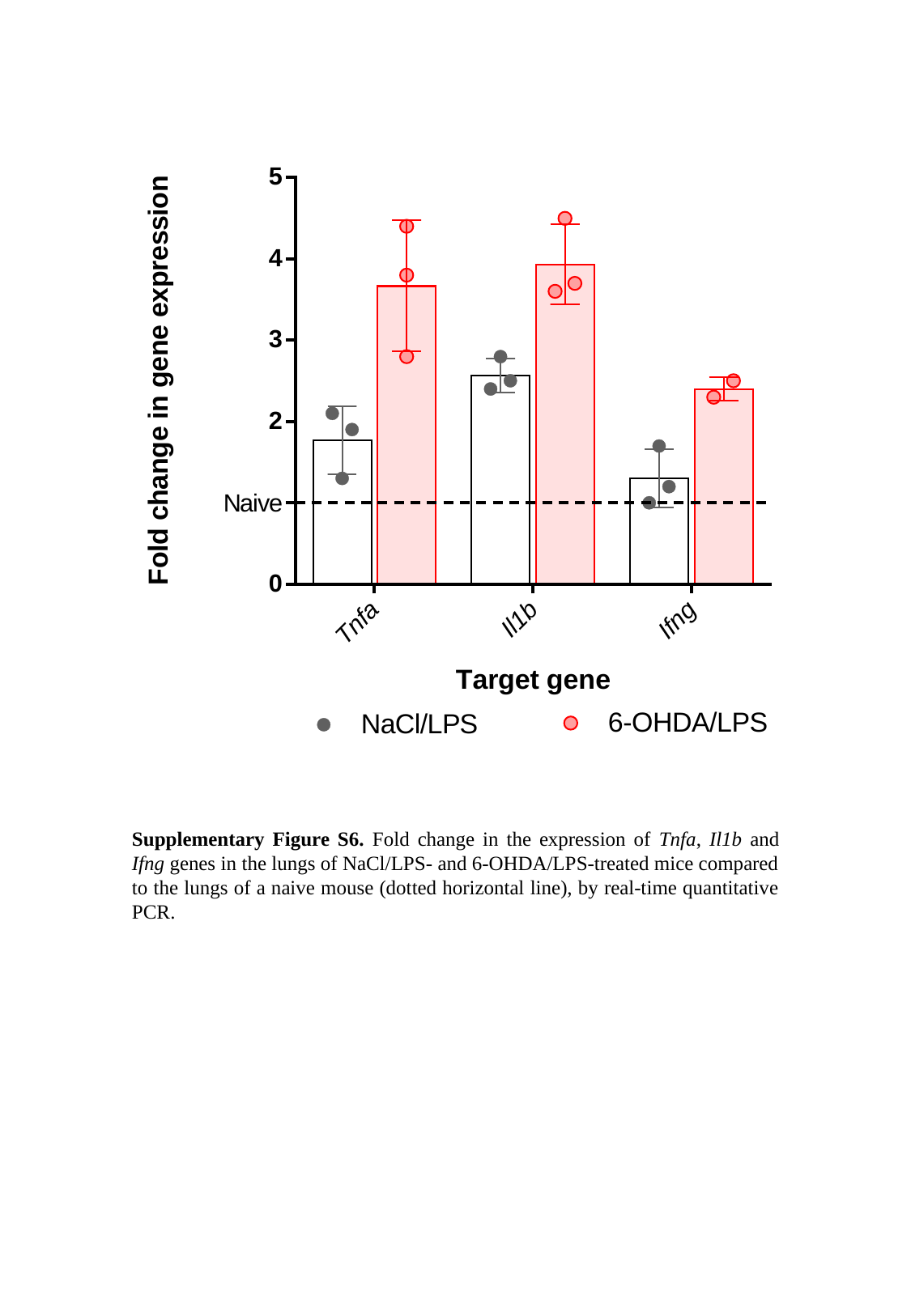

Supplementary Figure S6. Fold change in the expression of Tnfa, Il1b and Ifng genes in the lungs of NaCl/LPS- and 6-OHDA/LPS-treated mice compared to the lungs of a naive mouse (dotted horizontal line), by real-time quantitative PCR.

## Slide 8
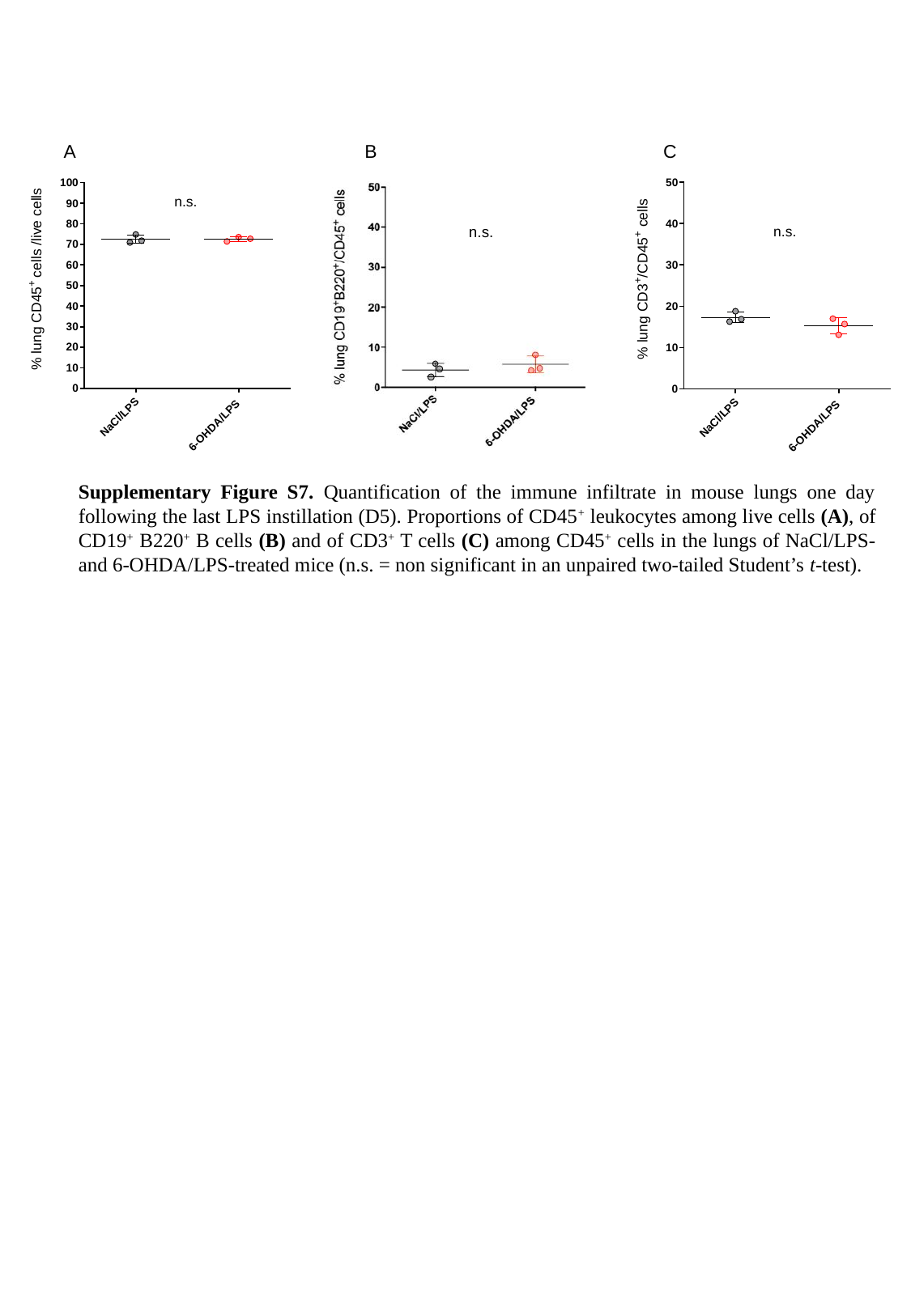

A
B
C
n.s.
Supplementary Figure S7. Quantification of the immune infiltrate in mouse lungs one day following the last LPS instillation (D5). Proportions of CD45+ leukocytes among live cells (A), of CD19+ B220+ B cells (B) and of CD3+ T cells (C) among CD45+ cells in the lungs of NaCl/LPS- and 6-OHDA/LPS-treated mice (n.s. = non significant in an unpaired two-tailed Student’s t-test).

## Slide 9
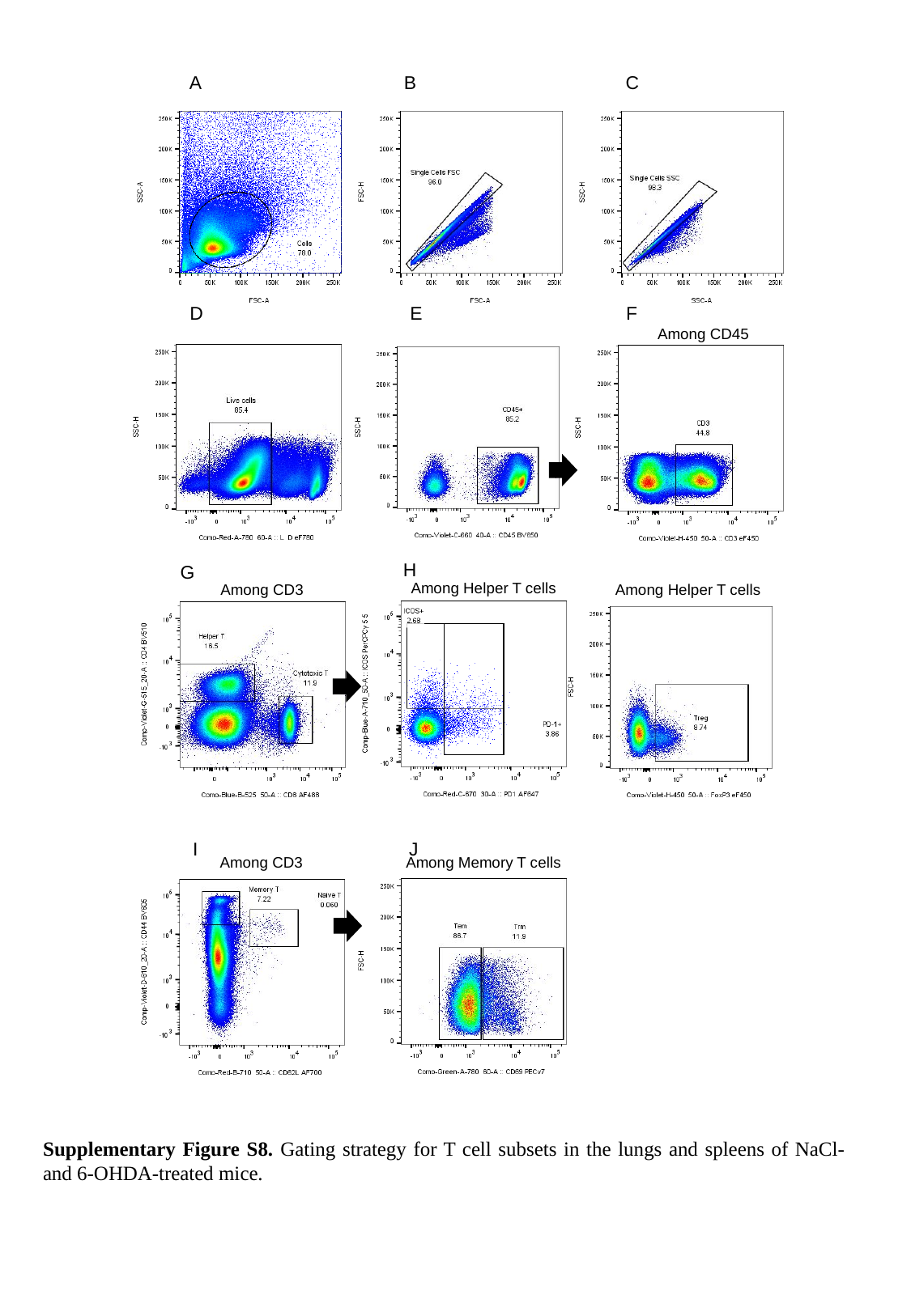

A
B
C
D
E
Among CD45
F
H
G
I
J
Among Helper T cells
Among CD3
Among Helper T cells
Among Memory T cells
Among CD3
Supplementary Figure S8. Gating strategy for T cell subsets in the lungs and spleens of NaCl- and 6-OHDA-treated mice.

## Slide 10
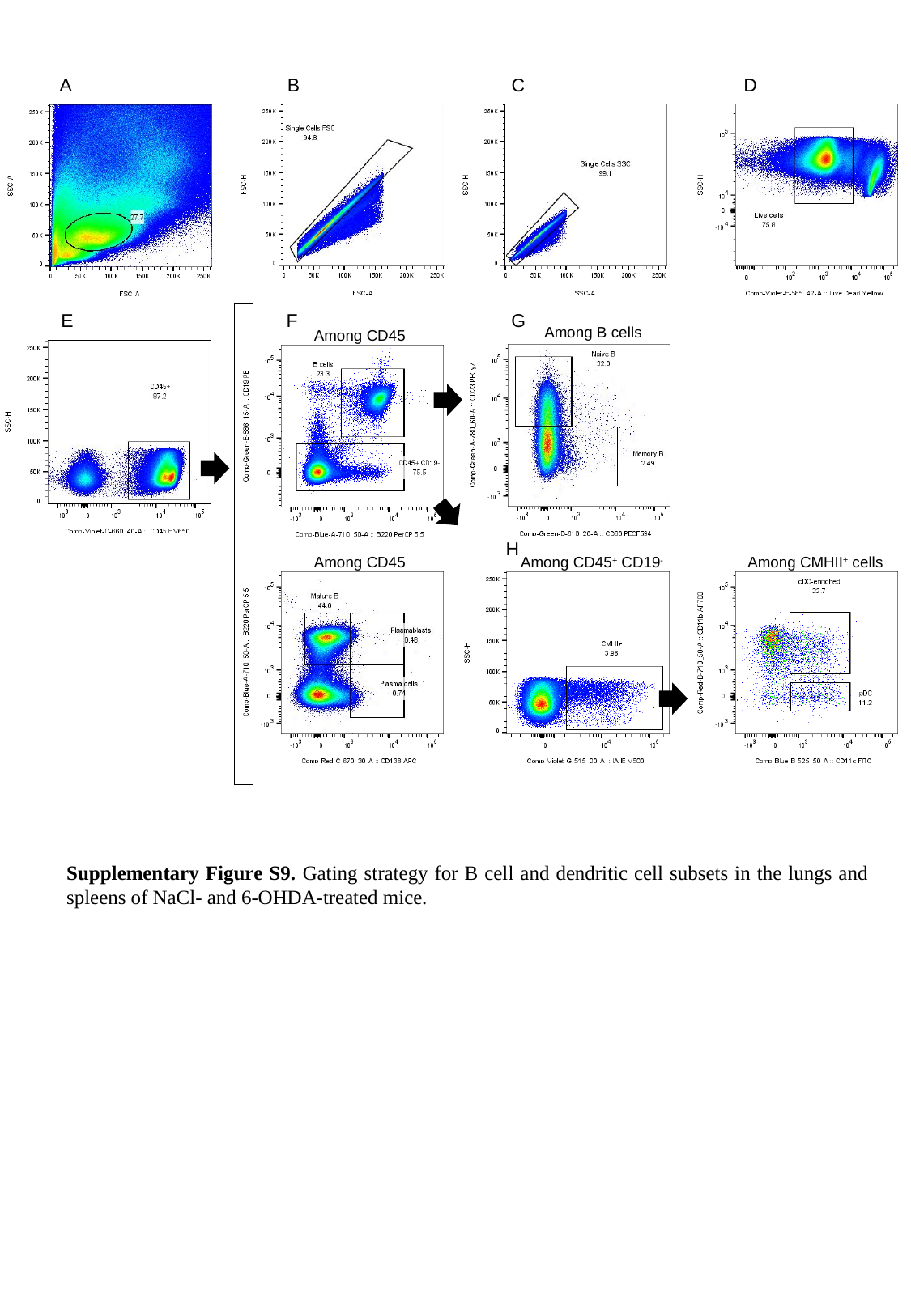

A
B
C
D
E
F
Among CD45
Among CD45
Among CD45+ CD19-
G
Among CMHII+ cells
Among B cells
H
Supplementary Figure S9. Gating strategy for B cell and dendritic cell subsets in the lungs and spleens of NaCl- and 6-OHDA-treated mice.

## Slide 11
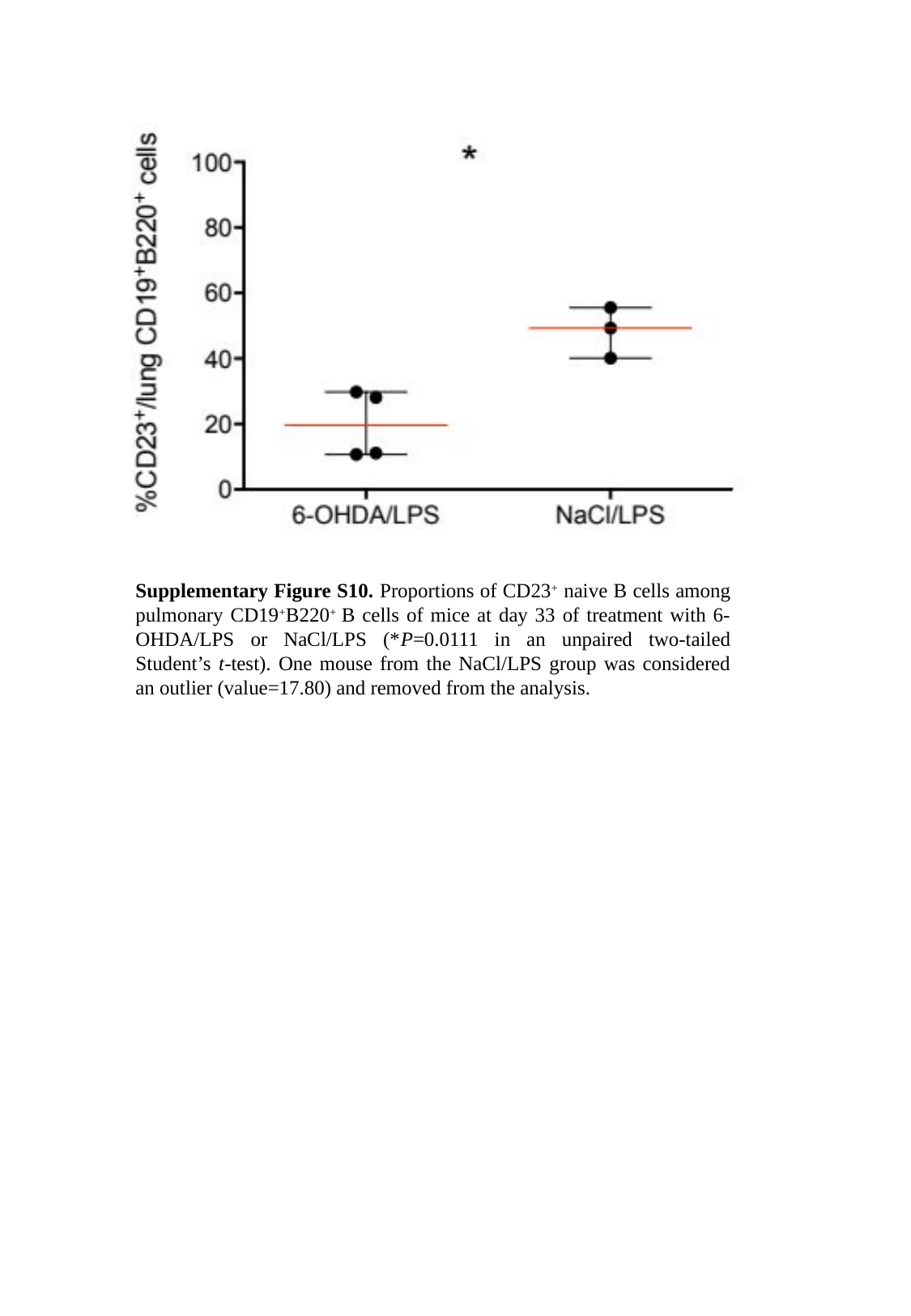

Supplementary Figure S10. Proportions of CD23+ naive B cells among pulmonary CD19+B220+ B cells of mice at day 33 of treatment with 6-OHDA/LPS or NaCl/LPS (*P=0.0111 in an unpaired two-tailed Student’s t-test). One mouse from the NaCl/LPS group was considered an outlier (value=17.80) and removed from the analysis.
