# Supplementary material for: Absence of sympathetic innervation hampers the generation of tertiary lymphoid structures upon acute lung inflammation
Source: Sci Rep. 2024 May 23;14:11749. doi: 10.1038/s41598-024-62673-0 (PMC11116507; doi:10.1038/s41598-024-62673-0)
Supplement: Supplementary file 2 — Supplementary Tables. [file 41598_2024_62673_MOESM2_ESM.pdf]

Supplementary Table S1. Antibodies and reagents used for immunofluorescence, western blot and ELISA.

Primary antibodies						
Target antigen	Host species and isotype	Clone	Manufacturer	Reference	Dilution	Unmasking pH
B220	Rat IgG2a	RA3-6B2	BioLegend	103202	1:1000	6.0
CD3	Rabbit IgG	SP7	Novus	NB600-1441	1:50	6.0
FDC	Rat IgG2c	FDC-M1	BD Pharmingen	551320	1:10	6.0
PNAd	Rat IgM	Meca-79	BD Pharmingen	553863	1:50	6.0
CD11c	Rabbit IgG	D1V9Y	Cell Signaling	97585S	1:100	9.0
FoxP3	Rat IgG2a	FJK-16s	eBioscience	14-5773-82	1:100	6.0
α-SMA	Mouse IgG2a	1A4	Sigma-Aldrich	C6198	1:200	N.A.[a]
Tyrosine hydroxylase	Rabbit IgG	N.A.	Sigma-Aldrich	AB152	1:200	N.A.
GAPDH	Rabbit IgG	N.A.	Invitrogen	TAB1001	1:3000	N.A.

Secondary antibodies					
Target species	Host species	Coupling	Manufacturer	Reference	Dilution
Rat IgG	Goat	HRP [b]	Eurobio	MP-7404-50	Ready-to-use
Rabbit IgG	Goat	HRP	Dako	K4003	Ready-to-use
Rat IgM	Goat	AF 647 [c]	Jackson Immunoresearch	115-605-075	1:400
Rat IgG	Goat	AF594	Invitrogen	A-11007	1:100
Rabbit IgG	Donkey	AF647	Invitrogen	A-31573	1:400

Tyramide and other fluorescent reagents			
Fluorochrome	Manufacturer	Reference	Dilution
DAPI	Thermo Scientific	62248	1:1000
CF430 [d]	Biotium	96053	1:100
CF514	Biotium	92199	1:62.5
AF555	Invitrogen	B40955	1:62.5
AF594	Invitrogen	B40957	1:62.5 to 1:32

(a) N.A.: Not Applicable
(b) HRP: HorseRadish Peroxidase
(c) AF: Alexa Fluor
(d) CF: Clear Fluor

Supplementary Table S2. Antibodies and reagents used for flow cytometry.

Target antigen	Host species and isotype	Clone	Fluorochrome	Manufacturer	Reference	Dilution
Antibodies						
CD45	Rat IgG2b	30-F11	BV650	BioLegend	103151	1:80
CD3e	Hamster IgG1	145-2C11	BV786	BD Biosciences	564379	1:50
CD3	Rat IgG2b	17A2	eF450	eBioscience	48-0032-82	1:25
CD4	Rat IgG2a	RM4-5	BV510	BD Pharmingen	563106	1:25
CD8a	Rat IgG2a	53-6.7	AF488	BioLegend	100723	1:50
CD44	Rat IgG2b	IM7	BV605	BioLegend	103047	1:12.5
CD62L	Rat IgG2a	MEL-14	AF700	eBioscience	56-0621-82	1:17
CD69	Hamster IgG1	H1.2F3	PE-Cy7	BD Pharmingen	552879	1:25
ICOS (CD278)	Hamster IgG	C398.4A	PerCP-Cy5.5	BioLegend	313518	1:25
PD-1 (CD279)	Rat IgG2b	RMP1-30	AF647	BD Pharmingen	566715	1:25
FoxP3	Rat IgG2a	FJK-16s	eF450	eBioscience	48-5773-82	1:167
CCR7 (CD197)	Rat IgG2a	4B12	PE	BD Pharmingen	560682	1:4
CD19	Rat IgG2a	1D3	PE	BD Biosciences	553786	1:25
B220 (CD45R)	Rat IgG2a	RA3-6B2	PerCP-Cy5.5	BD Pharmingen	552771	1:25
CD138	Rat IgG2a	281-2	APC	BD Biosciences	558626	1:25
CD23	Rat IgG2a	B3B4	PE-Cy7	BD Biosciences	562825	1:20
CD80	Hamster IgG2	16-10A1	PE-CF594	BD Biosciences	562504	1:20
CD11b	Rat IgG2b	M1/70	AF700	BD Biosciences	557960	1:50
CD11c	Hamster IgG1	HL3	FITC	BD Biosciences	557400	1:50
IA/IE	Rat IgG2b	M5/114.15.2	V500	BD Biosciences	562366	1:25
Reagents						
Live/Dead			eF780	Invitrogen	65-0865-18	1:25
Live/Dead			Yellow	Invitrogen	L34968	1:25

Supplementary Table S3. Total RNA were extracted from mouse lungs and cDNA was synthesized from 250-600 ng RNA using reverse transcriptase. Aliquots of cDNA were used as template for real-time qPCR reactions containing primers and probe for *Tnfa, Il1b, Ifng* or *Actb*. Each reaction contained cDNA derived from 25-60 ng total RNA. Each reaction was performed in two replicates.

		Ct				ΔCt (Average Target Ct – Average Actb Ct)			ΔΔCt (Average ΔCt – Average ΔCt Naive)			Normalized target (amount relative to Naive = $2^{-\Delta\Delta Ct}$)		
Group	Animal	*Tnfa*	*Il1b*	*Ifng*	*Actb*	*Tnfa*	*Il1b*	*Ifng*	*Tnfa*	*Il1b*	*Ifng*	*Tnfa*	*Il1b*	*Ifng*
Naive	1	27,93	23,87	30,66	20,43	7,50	3,44	10,22						
		28,03	23,92	30,62	20,54	7,48	3,38	10,07						
Average		*27,98*	*23,90*	*30,64*	*20,49*	*7,49*	*3,41*	*10,15*	*0,00*	*0,00*	*0,00*	*1,0*	*1,0*	*1,0*
SD		*0,07*	*0,04*	*0,03*	*0,08*	*-0,01*	*-0,04*	*-0,05*						
NaCl/LPS	1	24,34	20,09	27,39	18,59									
		24,70	20,29	27,59	17,58									
	2	24,63	19,78	27,87	17,63									
		25,23	20,22	28,24	18,09									
	3	23,49	18,90	26,65	17,15									
		24,17	19,41	27,70	17,36									
Average		*24,43*	*19,78*	*27,57*	*17,73*	*6,70*	*2,05*	*9,84*	*-0,80*	*-1,36*	*-0,31*	*1,7*	*2,6*	*1,2*
SD		*0,58*	*0,54*	*0,54*	*0,53*	*0,06*	*0,01*	*0,01*	*0,06*	*0,01*	*0,01*	*0,4*	*0,2*	*0,4*
6-OHDA /LPS	1	24,12	19,16	27,19	18,10									
		24,32	20,33	26,85	18,33									
	2	22,78	18,89	26,55	17,74									
		23,37	19,07	26,81	17,72									
	3*	23,08	18,99	25,03	17,43									
		23,26	19,33	25,27	17,74									
Average		*23,49*	*19,30*	*26,28*	*17,84*	*5,65*	*1,45*	*8,44*	*-1,85*	*-1,96*	*-1,71*	*3,6*	*3,9*	*2,4*
SD		*0,61*	*0,53*	*0,90*	*0,32*	*0,29*	*0,21*	*0,59*	*0,29*	*0,21*	*0,59*	*0,8*	*0,5*	*0,16*

* This mouse was considered an outlier for *Ifng* gene amplification data (normalized target amount = 6.0) and was therefore removed from the 6-OHDA/LPS group for statistical analyses.

Supplementary Table S4. Proportions of T cell, B cell and myeloid populations (mean value of data from 4 mice) in the spleen and lungs of NaCl/LPS- and 6-OHDA/LPS-treated mice at day 40.

	**Spleen**					**Lungs**				
	**6-OHDA**		**NaCl**		***t*-test°**	**6-OHDA**		**NaCl**		***t*-test°**
	Mean value (%)	Standard deviation	Mean value (%)	Standard deviation	***P*-value**	Mean value (%)	Standard deviation	Mean value (%)	Standard deviation	***P*-value**
$CD45^+$ cells/live cells	66.08	1.989	67.25	1.816	0.4164	74.87	6.152	77.20	4.834	0.5740
$CD3^+$ T cells/$CD45^+$ cells	32.15	2.838	28.58	2.675	0.1165	39.45	2.312	38.98	4.027	0.8446
$CD4^+$ helper T cells/T cells	15.45	0.885	13.43	1.475	0.0567	12.75	1.168	12.33	3.118	0.8070
$CD8^+$ cytotoxic T cells/T cells	12.90	1.675	11.85	1.162	0.3427	13.85	1.223	12.23	1.520	0.1468
$CD44^{hi}CD62L^-$ memory T cells/T cells	8.39	1.750	9.33	2.409	0.5521	33.13	5.482	38.35	5.087	0.2118
$CD69^-$ effector memory T cells/memory T cells	87.20	2.094	84.65	1.480	0.0939	87.40	1.985	83.18	4.005	0.1076
$CD69^+$ resident memory T cells/memory T cells	10.80	2.057	13.28	1.360	0.0912	11.10	1.901	15.03	3.879	0.1185
$CD44^{low}CD62L^+$ naive T cells/T cells	2.05	0.929	1.44	0.358	0.2682	5.58	2.402	3.89	2.524	0.3695
$PD\text{-}1^+$ cells/helper T cells	6.38	3.590	5.55	2.446	0.7163	3.02	0.870	6.21	3.251	0.1072
$ICOS^+$ cells/helper T cells	5.69	1.943	7.25	1.441	0.2425	16.03	4.703	22.35	13.34	0.4057
$FoxP3^+$ regulatory T cells/helper T cells	12.55	1.173	14.05	2.040	0.2496	5.55	1.450	5.35	0.538	0.7998
$CD19^+$ $B220^+$ B cells/$CD45^+$ cells [$]	88.55	1.644	84.55	5.193	0.1923	21.86	8.037	19.08	5.999	0.7862
$CD23^+$ naive B cells/$CD19^+$ $B220^+$ B cells [$]	89.00	2.535	90.95	1.139	0.2101	18.12	2.893	43.50	5.788	*0.0015**
$CD80^+$ memory B cells/$CD19^+$ $B220^+$ B cells	2.77	0.750	2.18	0.262	0.1849	3.82	1.248	2.60	0.285	0.1069
$CD138^+$ $B220^+$ plasmablasts/$CD45^+$ cells	1.52	0.695	2.34	0.548	0.1124	0.66	0.476	0.11	0.068	0.0607
$CD138^+$ plasma cells/$CD45^+$ cells	0.26	0.067	0.24	0.087	0.7266	0.39	0.235	0.10	0.052	*0.0497**
IA/IE^+ cells/$CD45^+$ $CD19^-$ cells	10.93	0.525	12.60	1.008	0.1911	2.09	0.102	2.42	0.225	0.2372
$CD11c^+$ plasmacytoid dendritic cells/IA/IE^+ cells	42.95	2.809	48.63	8.192	0.5366	10.25	1.091	16.18	0.948	*0.0063**
$CD11b^+$ $CD11c^+$ conventional dendritic cells-enriched cells/ IA/IE^+ cells	19.73	2.164	10.67	1.508	*0.0139**	24.45	1.559	24.38	1.224	0.9710

° unpaired two-tailed Student's *t*-test
* significant difference ($P<0.05$)
[$] analysis performed on 2 independent experiments (n=4/experiment)
