# Supplementary material for: Absence of sympathetic innervation hampers the generation of tertiary lymphoid structures upon acute lung inflammation
Source: Sci Rep. 2024 May 23;14:11749. doi: 10.1038/s41598-024-62673-0 (PMC11116507; doi:10.1038/s41598-024-62673-0)
Supplement: Supplementary file 5 — Supplementary Legends. [file 41598_2024_62673_MOESM5_ESM.docx]

**Legends to Supplementary videos**

**Video S1** Animated 3D rendering of α-SMA and TH staining in PFA-fixed mouse lung 40 days after NaCl intraperitoneal injections. α-SMA immunolabelling (red) shows the vascular network and TH immunolabelling (green) shows sympathetic nerve fibers. Staining was performed using iDISCO protocol. 3D rendering was obtained with IMARIS software.

**Video S2** Animated 3D rendering of α-SMA and TH staining in PFA-fixed mouse lung 40 days after 6-OHDA intraperitoneal injections. α-SMA immunolabelling (red) shows the vascular network and TH immunolabelling (green) shows only scattered dots and no fiber. Staining was performed using iDISCO protocol. 3D rendering was obtained with IMARIS software.
